# Proanthocyanidin biosynthesis in the developing wheat seed coat investigated by chemical and RNA‐Seq analysis

**DOI:** 10.1002/pld3.453

**Published:** 2022-10-12

**Authors:** Simon P. Vaughan, John M. Baker, Lucia F. Primavesi, Archana Patil, Robert King, Keywan Hassani‐Pak, Satish Kulasekaran, Jane Coghill, Jane L. Ward, Alison K. Huttly, Andrew L. Phillips

**Affiliations:** ^1^ Rothamsted Research Harpenden UK; ^2^ School of Biological Sciences University of Bristol Bristol UK

**Keywords:** flavonoids, gene function, grain color, proanthocyanidin, RNA‐Seq, wheat

## Abstract

The composition of proanthocyanidins in the testa (seed coat) of bread wheat was analyzed by thiolysis of PA oligomers from developing grain and found to consist of (+)‐catechin monomers, with a small amount of (+)‐gallocatechin. The average chain length of soluble PA stayed relatively constant between 10 and 20 days post‐anthesis, whereas that of unextractable PA increased over the same period, suggesting that increases in chain length might account for the insolubility of PAs from mature wheat grain. We carried out RNA‐Seq followed by differential expression analysis from dissected tissues of developing grain from red‐ and white‐grained near‐isogenic lines differing in the presence of an active *R* gene that encodes a MYB transcription factor involved in control of PA biosynthesis. In addition to genes already identified encoding chalcone synthase, chalcone isomerase, flavanone 3‐hydroxylase, and dihydroxyflavonoid 4‐reductase, we showed that wheat genes encoding phenylalanine ammonia lyase, flavonoid 3′,5′‐hydroxylase, leucoanthocyanidin reductase, and a glutathione *S*‐transferase (the orthologue of maize *Bronze‐2*) were more highly expressed in the red NIL. We also identified candidate orthologues of other catalytic and regulatory components of flavonoid biosynthesis in wheat.

## 1 INTRODUCTION

The true seed coat (testa) of many plant species contains proanthocyanidins (PAs), polymeric flavonoids also known as condensed tannins that acquire a reddish color on oxidation. There is evidence from several species, including Arabidopsis and wheat (*Triticum aestivum*), that seed coat PAs contribute to seed dormancy: seeds of mutants that fail to accumulate PA in the testa germinate more readily than wild‐type lines (Debeaujon et al., 2000; Himi et al., 2002). Thus, white‐grained lines of wheat, which lack PAs, are sensitive to environmental conditions during grain development that result in low embryo dormancy, leading to pre‐harvest sprouting (PHS) that reduces grain quality and economic value (Torada & Amano, 2002). For some food uses, such as noodles, white‐grained varieties are preferred, but their environmental sensitivity makes them unsuitable for cultivation in regions with high rainfall and humidity in the growing season, including maritime climates such as the United Kingdom. As part of a wider strategy to develop red and white wheat varieties with increased PHS resistance, we aim to understand how seed‐coat PAs promote dormancy; a prerequisite is a more complete understanding of the control of PA biosynthesis in wheat, which is currently lacking.

**FIGURE 1:**
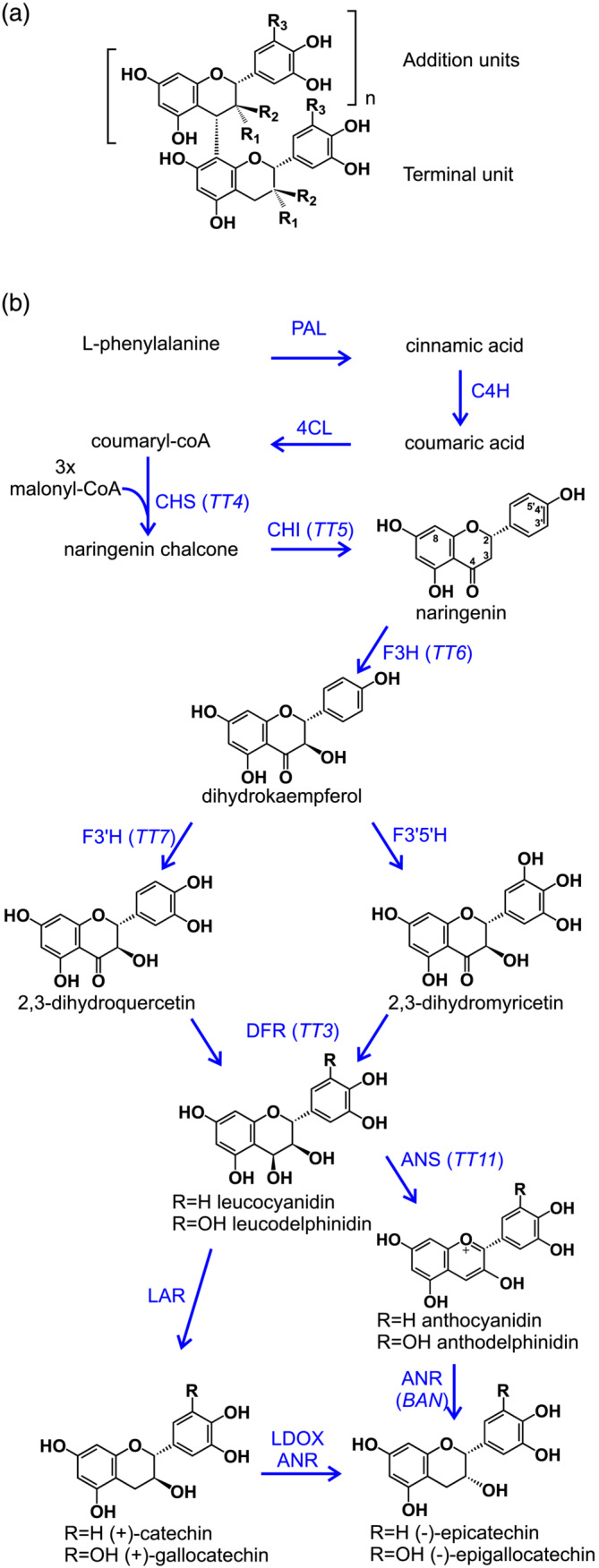
Proanthocyanidin (PA) structure and biosynthesis. (a) Generalized structure of oligomeric PA. Subunits are (+)‐catechin (R_1_ = H, R_2_ = OH, R_3_ = H); (−)‐epicatechin (R_1_ = OH, R_2_ = H, R_3_ = H); (+)‐gallocatechin (R_1_ = H, R_2_ = OH, R_3_ = OH); (−)‐epigallocatechin (R_1_ = OH, R_2_ = H, R_3_ = OH). (b) Simplified biosynthetic pathway from phenylalanine to the terminal flavan‐3‐ol subunits. PAL, phenylalanine ammonia lyase; C4H, cinnamate 4‐hydroxylase; 4CL, 4‐coumaroyl coA ligase; CHS, chalcone synthase; CHI, chalcone isomerase; F3H, flavanone 3‐hydroxylase; F3′H, flavonoid 3′‐hydroxylase; F3′5′H, flavonoid 3′,5′‐hydroxylase; DFR, dihydroxyflavonoid 4‐reductase; ANS, anthocyanidin synthase; LDOX, leucoanthocyanidin dioxygenase; ANR, anthocyanidin reductase; LAR, leucoanthocyanidin reductase

PAs are oligomers or polymers of flavan‐3‐ols, typically (+)‐catechin and (−)‐epicatechin; PA from some species may also contain gallocatechin or epigallocatechin subunits (with both 3′ and 5′ hydroxylation in the B‐ring) or, more rarely, afzelechin or epiafzelechin (with neither 3′ nor 5′ hydroxyls). Oligomeric PAs consist of a terminal subunit with a variable number of C4 → C8‐linked addition units (Figure 1a); across the plant kingdom, the most common terminal unit is catechin, and the most common addition unit is epicatechin (Dixon & Sarnala, 2020), but this statistic conceals a high degree of variation between species. For example, Arabidopsis PA is exclusively composed of epicatechin (Abrahams et al., 2003), whereas barley PA is reportedly a polymer of catechin, with little epicatechin detected (Jun et al., 2021) and grape seed PAs consist of a complex mixture of oligomers containing catechin, epicatechin, and epicatechin gallate subunits (Sharma et al., 2011).

Although the biosynthetic pathway from the flavonoid precursor naringenin to the flavan‐3‐ol monomers appears straightforward (see simplified scheme in Figure 1b), the reality of oligomeric PA biosynthesis is more complex. It appears clear that the catechin monomers, (+)‐catechin or (−)‐epicatechin, only contribute the terminal unit of PA; furthermore, the biosynthetic route to these terminal units differs between species. In species such as *Desmodium uncinatum* or barley, catechin is formed by the action of leucoanthocyanin reductase (LAR) on 2,3‐trans‐leucoanthocyanidin. Arabidopsis lacks LAR and therefore produces epicatechin as terminal subunits by the sequential action of anthocyanidin synthase (ANS) and anthocyanidin reductase (ANR) on 2,3‐trans‐leucoanthocyanidin (Figure 1b); there is evidence that the extension units are carbocations derived from cyanidin (Wang et al., 2020). In contrast, *Medicago trunculata*, whose PA, like Arabidopsis, is wholly composed of epicatechin subunits, has a functional LAR but does not accumulate catechin as this is substrate for a homologue of ANS that produces anthocyanidin, converted to (−)‐epicatechin by the action of ANR (Jun et al., 2018). The most likely source of catechin extension units is 2,3‐trans‐leucoanthocyanidin, possibly in the form of a carbocation, whereas in Medicago, the likely addition unit is derived from 2,3‐cis‐leucoanthocyanidin, formed by ANS and a secondary activity of ANR (Jun et al., 2021).

PA biosynthesis is controlled at least in part by transcriptional regulation of the genes in the pathway. In dicots such as Arabidopsis, distinct transcription factors are involved in controlling the early biosynthetic genes (EBGs: chalcone synthase [*CHS*], chalcone isomerase [*CHI*], flavanone 3‐hydroxyase [*F3H*], and flavonoid 3′‐hydroxylase [*F3′H*]) (Stracke et al., 2007) versus the late biosynthetic genes (LBGs: dihydroflavonol reductase [*DFR*], *ANS*, *ANR*, and *TT12*) (Nesi et al., 2001). Although the Arabidopsis EBGs are controlled by a set of Myb transcription factors, including MYB11, MYB12, and MYB111 (Stracke et al., 2007), expression of the LBGs in seeds is mediated by a complex of three transcription factors: a Myb (TT2), a bHLH (TT8), and a WD40‐repeat protein (TTG1) (Baudry et al., 2004). In wheat, only the orthologue of *TT2* (*R*, for *Red*) has been characterized (Himi, Maekawa, Miura, & Noda, 2011): loss of function mutants lacking functional copies of all three homoeologues of the *R* gene (encoding TaMyb10) on the group 3 chromosomes lack seed PAs and are classified as white‐grained (note that the wheat Myb *R* is unrelated to the maize *R* gene family that encode bHLH transcription factors). Only one wild‐type *R* allele is required to confer a red‐grained phenotype in wheat, although there is a small gene‐dosage effect (Bassoi & Flintham, 2005). In contrast to Arabidopsis, the wheat R/TaMyb10 transcription factor appears to promote expression of not only *DFR* from the LBG group (Himi et al., 2005) but also *CHS*, *CHI*, and *F3H*, members of the EBG group. However, the genes encoding the later biosynthetic steps in PA biosynthesis in wheat have yet to be identified.

In the work presented below, we investigate the location and composition of wheat PAs and use RNA‐Seq analysis of dissected tissues of developing grain of red and white isolines to identify additional genes controlled, directly or indirectly, by R/TaMyb10 that are candidates for involvement in PA biosynthesis.

## 2 MATERIALS AND METHODS

### 2.1 Plant materials and growth conditions

Near‐isogenic lines of the white‐grained Holdfast cultivar of bread wheat (*T. aestivum* L.), which is null for all homoeologues of *R* (genotype *R‐A1a*/*R‐B1a*/*R‐D1a*), containing an introgressed (to BC_6_) wild‐type allele, *R‐D1b*, derived from cv. Chinese Spring (Bassoi & Flintham, 2005) (“Holdfast‐Red”) were supplied by Dr. John Flintham, JIC, Norwich, UK. Seeds were germinated on damp filter paper, and seedlings in compost were vernalized for 8 weeks at 8°C before transfer to 15 cm pots of Rothamsted prescription mix compost with added nutrients (75% medium grade peat, 12% sterilized loam, 3% medium grade vermiculite, 10% lime‐free grit, 3.5 g L^−1^ “Osmocote Exact 3–4 month” [Scotts Ltd., Godalming, Surrey], 0.5 g L^−1^ PG mix [Hydro Agri Ltd., Bury St. Edmunds, Suffolk], lime [approximately 3 g L^−1^] to pH 5.5–6.0, Vitax Ultrawet 0.2 ml L^−1^). Plants were grown under daylight with supplementary lighting to 250 μmol m^−2^ under a 16 h day^−1^. Mature grains of barley cv. Golden Promise were obtained from field‐grown material.

### 2.2 Light imaging

For DMACA staining of developing grains, transverse portions of the central region of grains at 15 days post‐anthesis (dpa) were frozen to a stub in OCT (Tissue‐Tek: Sakura Finetek USA) in liquid N_2_. Sections, 30 μm, were cut on a Leica CM1850 cryostat and pressed on to polylysine slides before being stained in DMACA (1% DMACA [4‐dimethylaminocinnamaldehyde, Sigma] in 0.7 M HCl) for 1–2 min (Li et al., 1996). All images were taken using a Zeiss Axiophot microscope and MetaMorph imaging software (Molecular Devices LLC, San Jose, CA, USA).

### 2.3 Analytical chemistry

For analysis of PAs in developing grain, ears of red‐ and white‐grained NILs of wheat cv. Holdfast were tagged at anthesis and harvested at 10, 15, and 20 days post‐anthesis (dpa) as well as at maturity (>60 dpa); material was freeze dried before analysis. For analysis of oligomeric PAs, 15–100 mg of ground, dried material was extracted with 70% acetone, separated on Sephedex LH‐20 in a Pasteur pipette and analyzed by electrospray‐ionization mass spectroscopy (ESI‐MS, Bruker Esquire 3000). To determine the monomer composition and stereochemistry, in situ thiolysis was performed on 5 mg dry tissue by treatment with 100 μl of 5% benzyl mercaptan in methanol (Gu et al., 2002), adding 50 μl 3.3% HCl in methanol, then heating at 40°C for 1 h, with mixing (950 rpm). Extended incubations with standards confirmed that these conditions did not promote epimerization of the products. The supernatant was separated after centrifugation (5 min), and aliquots of 10 μl were analyzed by reverse‐phase HPLC (Agilent 1100) on an Ascentis C18 column maintained at 25°C using a methanol (A) vs water (containing 2% acetic acid) (B) gradient (0–5 min: 20% A, 15 min: 35% A, 20 min: 50% A, 40 min: 65% A, 45–50 min: 100% A.), with detection at 280 nm. The flow rate was 1 ml min^−1^. Products were quantified using a calibration curve based on catechin with relative extinction coefficients. PA concentration was calculated as the sum of the amounts of the terminal catechins/gallocatechins and their benzylthioethers. The mean degree of polymerization (average chain length) was calculated as the ratio of the benzythioethers to the terminal catechins. Statistical comparisons between samples were carried out using ANOVA and Student's *t*‐test.

To characterize the products from heterologous expression in yeast or *E. coli*, samples were analyzed by high resolution LC–MS in negative ion mode. An aliquot (500 μl) of culture was removed to a clean tube, and after centrifugation (13,500 rpm, 5 min), the supernatant (400 μl) was transferred to a glass vial for LC–MS analysis. LC–MS were recorded using an LTQ‐Orbitrap Elite mass spectrometer (Thermo Fisher Scientific) coupled to a Dionex Ultimate 3000 RS UHPLC system. Chromatographic separation was carried out at 35°C using a reversed phase Hypersil Gold column (1.9 μm, 30 × 2.1 mm i.d. Thermo Fisher Scientific, Germany). Separation solvents consisted of water/0.1% formic acid (A) and acetonitrile/0.1% formic acid (B), and the chromatographic run was 25 min under the following conditions: 0 min, 5% B; .1–20 min, 5%–100% B; 20–25 min. The injection volume was 10 μl, and the flow rate was set to 0.3 ml/min. Mass spectra were collected using a heated ESI source and were acquired in negative mode with a resolution of 120,000 over m/z 50–1500. The source voltage, sheath gas, auxiliary gas, sweep gas, and capillary temperature were set to 2.5 kV, 35 (arbitrary units), 10 (arbitrary units), 0.0 (arbitrary units) and 350°C, respectively. Automatic MS–MS was performed on the top four ions using an isolation width of m/z 2. Selected ions were fragmented using HCD with a collision energy of 65 and an activation time of 0.1 ms. Peaks of interest were integrated within Xcalibur v. 2.2 software.

### 2.4 RNA‐Seq analysis

For transcript analysis, grains from red and white isolines were harvested at 12 days post‐anthesis. After removal of the embryo, grains were separated by hand dissection into endosperm (E), inner pericarp (IP; consisting of the aleurone, nucellar epidermis, inner and outer integuments, any remaining tube cells, and cross cells) and outer pericarp (OP; consisting of the mesocarp parenchyma, hypodermis, and epidermis) (Pearce et al., 2015) and frozen in liquid N2. Three biological replicate samples were collected for each tissue. RNA was extracted, Illumina Tru‐Seq libraries prepared and single end 110 bp reads generated as described previously (Pearce et al., 2015). Reads were mapped to the IWGSCv1.0 bread wheat (cv. Chinese Spring) assembly and the RefSeq v1.1 GTF with high and low quality gene models (Appels et al., 2018), obtained from Ensembl Plants (http://plants.ensembl.org/Triticum_aestivum/), using HISAT2 (Kim et al., 2015) (version 2.3.3) on the Galaxy platform (Afgan et al., 2018); featureCounts (Liao et al., 2014) (Galaxy version 1.5.3) and DESeq2 (Love et al., 2014) (Galaxy version 2.11.38) were used to identify differentially expressed transcripts at a false discovery rate (FDR) of 0.05. StringTie (Pertea et al., 2015) (Galaxy version 1.2.3) was used to identify un‐annotated transcripts and to calculate tpm (transcripts per million) counts. Differentially expressed genes were annotated using Blast2GO (BioBam Bioinformatics, Valencia, Spain) and potential functions assessed using the gene discovery software KnetMiner version 3.2 (http://knetminer.rothamsted.ac.uk/; Hassani‐Pak et al., 2016) and the Wheat Knowledge Graph, release 45 (Hassani‐Pak et al., 2016). Unaligned RNA‐Seq reads have been deposited in the European Nucleotide Archive (http://ebi.ac.uk/ena) with accession number E‐MTAB‐9006.

### 2.5 Heterologous expression in yeast and *E. coli*

Synthetic cDNAs encoding candidate flavonoid 3′‐ and 3′,5′‐hydroxylases (TraesCS1A01G442300 and TraesCS6B01G405900) were codon‐optimized for yeast, synthesized and inserted as BamHI‐HindIII fragments into plasmid pESC‐Leu (Genscript Biotech, the Netherlands). The recombinant plasmids, with pESC‐Leu as negative control, were introduced into *Saccharomyces cerevisiae* strain WAT11 (W303‐1B; MATa; *ade2‐1*; *his3‐11,15*; *leu2‐3*; *trp1‐1*), which overexpresses the Arabidopsis cytochrome P450 reductase (Pompon et al., 1996), and the resultant yeast lines grown to log phase at 30°C in YNB‐Leu dropout medium (Sigma) supplemented with adenine (80 mg/L) and 2% glucose. The cells were pelleted by centrifugation and resuspended to an OD_600_ of 1.0 in the same medium supplemented with 2% galactose, replacing glucose to induce protein production, and were incubated shaking at 28°C overnight. The cells were again pelleted and resuspended in dropout medium with galactose to an OD_600_ of 1.0, and naringenin or eriodictyol in DMSO were separately added at final concentrations of 100 μM to 10 ml aliquots of cells in 250 ml conical flasks. Samples of culture were analyzed after 24 h shaking at 28°C.

For functional characterization of one homoeologue of the candidate LAR gene, TraesCS4D01G218700, the open reading frame was codon‐optimized for *E. coli*, synthesized (Genscript) and inserted in pETM6‐16 (Addgene) under a T7 promoter. pETM6‐16 contains codon‐optimized cDNAs for *Camellia sinensis* flavanone 3‐hydroxylase (F3H) and *Fragaria ananassa* dihydroflavonol 4‐reductase (DFR), each also under a T7 promoter (Zhao et al., 2015). The resulting plasmid, pETM6‐16‐TaLAR, was introduced into *E. coli* strain Rosetta‐2 (Novagen) in parallel with negative (pETM6‐16) and positive (pETM6‐168, consisting of pETM‐16 with a LAR coding region from *Desmodium uncinatum*) *(*Zhao et al., 2015 *)* control plasmids. Log phase cultures, 2 ml, of *E. coli* carrying each plasmid in 2xYT medium were induced by addition of IPTG to 1 mM. Eriodictyol and dihydroquercetin in DMSO were separately added to 100 μM, and the cultures were incubated, shaking, for 45 h at 30°C before sampling for analysis.

### 2.6 Phylogenetic analysis

For phylogenetic analysis, wheat protein sequences related to the target were identified by BLAST search to IWGSCv1.0 within Geneious v10.6 (Biomatters Ltd.). Related sequences from other species were identified by BLAST at Phytozome (https://phytozome.jgi.doe.gov/pz/portal.html). Protein sequences were aligned using MUSCLE, and after optimizing the alignment, amino acid positions containing gaps were removed. Phylogenetic analysis was carried out using RaxML on the CIPRES Science Gateway (www.phylo.org) after optimization using TOPALi v2.5 (Milne et al., 2009); trees were displayed using MEGA6 (Tamura et al., 2013).

## 3 RESULTS

### 3.1 Analysis of PAs from wheat and barley grain

**FIGURE 2:**
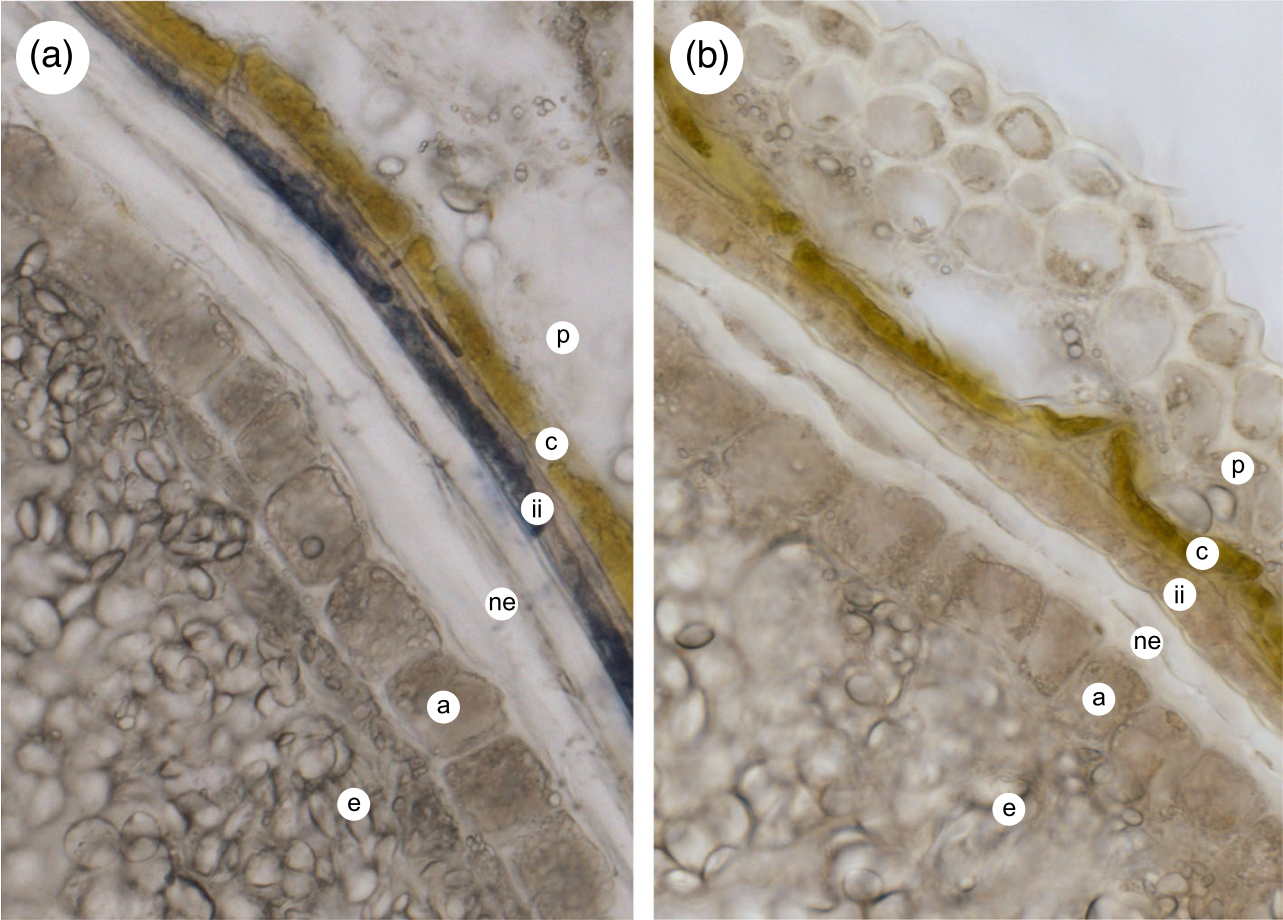
Cross‐section of developing wheat grain showing DMACA‐stained PA accumulation in the inner integument cells of (a) red‐ and (b) white‐grained (negative control) lines at 15 days post‐anthesis. p, pericarp; c, cross cells containing chloroplasts; ii, inner integument layers; ne, nucellar epidermis; a, aleurone; e, endosperm. Scale bar = 50 μm

As reported recently by Kohyama and colleagues (Kohyama et al., 2017), staining of mature barley caryopses with dimethylaminocinnamaldehyde (DMACA) in acidic methanol, which reacts with monomeric and polymeric flavan‐3‐ols (Peng et al., 2012), resulted in blue staining of the testa and the solubilization of substantial amounts of staining material into the medium. In contrast, treatment of mature wheat caryopses with DMACA resulted in staining only of material in the testa, with no release of stained compounds into the medium, implying that the proanthocyanidins present in mature wheat grain are not easily solubilized. Jun and colleagues similarly failed to extract any soluble PA from mature wheat grain (Jun et al., 2021). To study this further, we carried out analysis of soluble PAs and precursors from bran material from mature barley and red‐grained bread wheat. ESI‐MS analysis of partially purified 70% acetone extracts of barley bran revealed compounds with m/z values consistent with PA oligomers from dimers to hexamers (Figure S1); in contrast, similar extracts from mature red wheat bran contained no detectable PA oligomers. However, 70% acetone extracts from developing wheat grain at 15 days post‐anthesis did contain PA oligomers (Figure S1), and staining of developing grain sections with DMACA showed that this material accumulated in the inner layer of the inner integument of red‐grained lines, but staining was absent from white‐grained lines (Figure 2).

An analysis of PA monomers and dimers in developing wheat grain (10–15 dpa) by Kohyama et al. (2017) identified the presence of (+)‐catechin, procyanidin B3 (catechin‐(4α → 8)‐catechin dimer), and prodelphinidin B3 (gallocatechin‐(4α → 8)‐catechin heterodimer) in 75% acetone extracts. We analyzed the monomer composition and chain length of PA oligomers at different times during early wheat grain development. Acid‐catalyzed depolymerisation of PAs in the presence of a nucleophile such as benzylmercaptan results in release of the terminal subunit in its native state (e.g., catechin or gallocatechin) together with benzylthioether (BTE) derivatives of the extension subunits (Figure S2), thus allowing determination of the mean chain length of the substrate PA. In contrast to simple acid depolymerisation, which yields cyanidins, thiolysis retains the stereochemistry at C2‐C3 and therefore allows identification of both (gallo)catechin and (gallo)epicatechin subunits of the oligomer. Note that extension units of catechin yield a mixture of stereoisomers of the BTE group at C4 whereas epicatechin extension units yield only 3,4‐*trans*‐epicatechin‐BTE (Figure S2).

**FIGURE 3:**
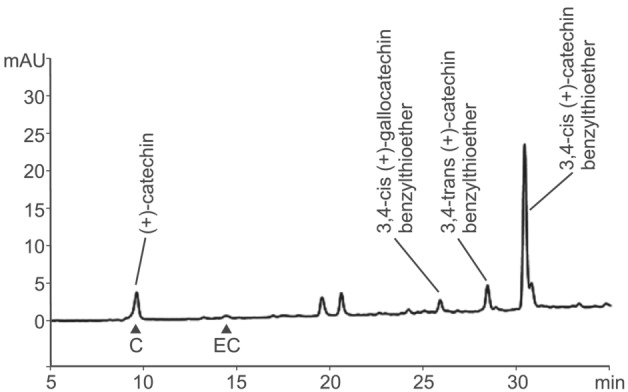
HPLC separation of thiolysis products of wheat PAs after reaction with benzylmercaptan in acidic methanol showing (+)‐catechin terminal unit and benzylthioether conjugates of (+)‐catechin and (+)‐gallocatechin extension units. The elution times of (+)‐catechin (C) and (−)‐epicatechin (EC) standards are indicated.

**FIGURE 4:**
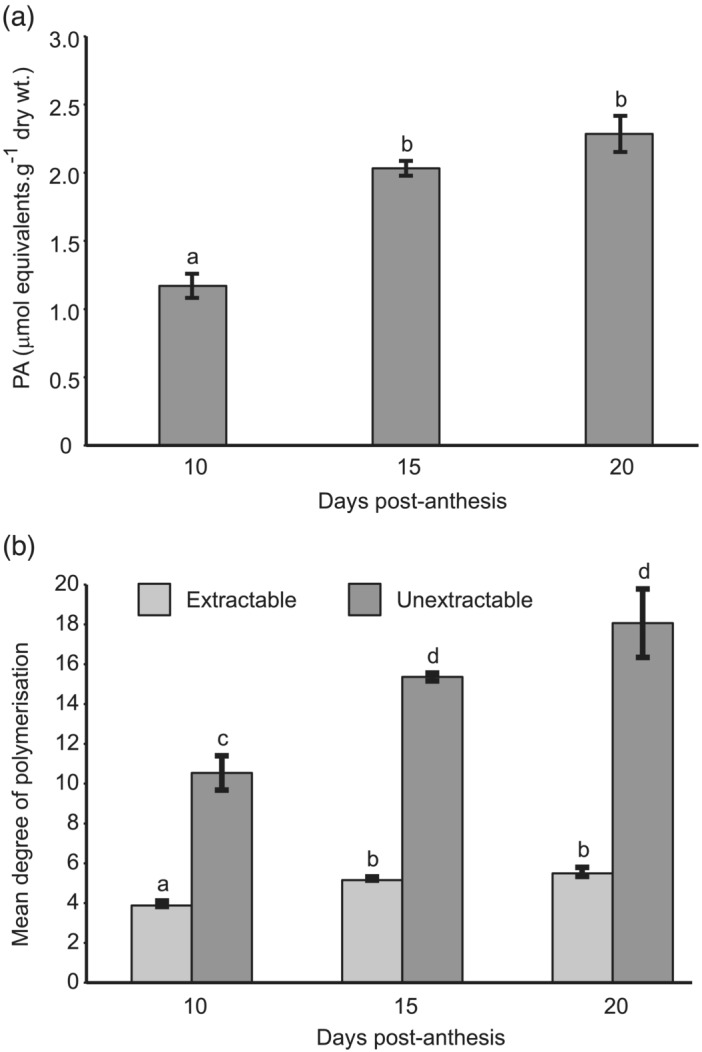
Quantification of PA content and degree of polymerization in grain development. (a) Total PA content and (b) degree of polymerization (DP) of PA extracted from grain of red lines during development. Standard error of the means of three replicates is indicated for each sample; letters above bars indicate significant differences between samples (*p* < .05).

An example of benzylmercaptan thiolysis of acetone‐soluble PAs extracted from wheat grain at 15 days post‐anthesis (dpa) and separated by reverse‐phase HPLC conditions that clearly resolved (+)‐catechin and (−)‐epicatechin is shown in Figure 3. The terminal and BTE‐linked extension units detected were all derived from (+)‐catechin, with a small proportion of (+)‐gallocatechin; no (−)‐epicatechin or (−)‐epigallocatechin terminal or extension units were detected. As the method was shown to be successful on both soluble and insoluble PAs, it was possible to estimate the amount of total PA and also, by in situ thiolysis of the residue after extraction with 70% acetone, the concentration of insoluble PAs. Figure 4a shows the results of estimating PA content of developing grains of red grained wheat (cv. Holdfast) at various times after anthesis. Over the period between 10 and 15 days post‐anthesis, the total PA content increased from 1.2 to 2.3 μmol monomer catechin equivalents per g dry weight; a small but statistically insignificant increase in PA content was detected between 15 and 20 dpa. Over the same period, the mean degree of polymerization (DP; average chain length) of unextractable PAs in the developing grain rose from 1.5 to 18.1 (Figure 4b), whereas the mean DP of soluble forms stayed relatively constant at 3.9 to 5.5. This demonstrates that the longer chain PAs are not extracted in 70% acetone, suggesting that increasing chain length during grain development may be responsible for the lack of extractable PAs in mature grain.

### 3.2 Identification of genes expressed in red versus white developing wheat grain tissues

As discussed above, red‐grained varieties of wheat possess at least one functional copy of *R* (*TaMyb10*) whereas white‐grained lines are null for both copies of all three homoeologues (Himi, Maekawa, Miura, & Noda, 2011). By analogy with other flavonoid biosynthetic pathways, R/TaMyb10 acts, probably in association with other transcription factors, to control expression of a subset of the PA biosynthetic genes: analysis of the expression of *CHS*, *CHI*, *F3H* and *DFR* in developing grain of red (ANK‐1C; R‐D1a/R‐B1a/R‐D1b) and white (Novosibirskaya‐67; R‐D1a/R‐B1a/R‐D1a) near‐isogenic lines has shown that homologues of all four genes are more highly expressed in the former than the latter (Himi et al., 2005). In dicots, genes later in the pathway are similarly controlled by Myb transcription factors, suggesting that other genes in the wheat PA pathway that are directly or indirectly controlled by *R/TaMyb10* could be identified by comparing the transcriptome of red versus white isolines. We therefore carried out RNA‐Seq analysis of developing grain of red and white NILs of bread wheat cv. Holdfast. Sample were collected at 12 dpa as this precedes the peak of PA biosynthesis (Figure 4a). To increase sensitivity of detection of differentially expressed genes, the “inner pericarp” (IP) layer was separated, as far as possible, from the outer pericarp (OP) and endosperm (E) tissues by hand dissection. All three tissues were investigated by RNA‐Seq to identify genes differentially expressed in the tissue/genotype combination that accumulates PA, that is, the inner integument within the IP tissue of red lines that express a functional R Myb (Figure 2).

Single‐end RNA‐Seq reads from dissected OP, IP and E tissues of developing grain from red and white lines were mapped to reference transcript sequences from bread wheat cv. Chinese Spring (Appels et al., 2018). The RNA‐Seq data from red isolines within this dataset have been previously analyzed and demonstrated, using the expression pattern of marker genes, that the three tissues types had been reasonably well separated by dissection, with a small amount of contamination of endosperm (E) tissues with IP and vice versa (Pearce et al., 2015). Figure S3 shows MA‐plots for the mapping to the IWGSCv1.0 transcriptome using the HISAT2‐featureCounts‐DESeq2 workflow. This identified 69 differentially expressed genes (DEGs) that were significantly more highly expressed (*p*_adj_ < .05) in red IP than in white, and 76 more highly expressed in white than red IP (Table S2). Potential identities and functions of the DEGs were determined using Blast2GO and KnetMiner (Hassani‐Pak et al., 2016) which found 25 genes, representing 10 gene families, with established roles in flavonoid biosynthesis or its regulation in plants, that were significantly up‐regulated in red grain (Tables 1 and S4). Note that due to the difficulty of isolating dissected tissues from immature grains, the low yield of RNA precluded confirmation of differences in gene expression by QRT‐PCR; however, in most cases, the RNA‐Seq data revealed that all three homoeologues of the identified genes showed differential expression (Table 1), increasing confidence in the results.

**TABLE 1:** Wheat genes involved in PA biosynthesis identified as being differentially expressed in developing IP tissues of red and white NILs of cv. Holdfast

ID	Gene ID (IWGSCv1.0)	Mean TPM		DEseq2	
		IP‐R	IP‐W	*p*‐adj (IP)	FC (IP)
PAL	TraesCS2A02G380800	1.8	0.0	2.4 E‐04	3.1
PAL	TraesCS2B02G398000	9.6	1.8	5.6 E‐11	3.8
PAL	TraesCS2D02G377200	1.8	0.1	4.4 E‐03	2.6
CHS (TT4)	TraesCS2A02G025700	7.5	0.1	2.8 E‐12	5.8
CHS (TT4)	TraesCS2A02G527700	16.2	0.1	2.8 E‐27	11.7
CHS (TT4)	TraesCS2B02G038700	12.4	1.1	2.8 E‐13	5.4
CHS (TT4)	TraesCS2B02G558400	12.1	0.0	3.5 E‐25	10.9
CHS (TT4)	TraesCS2D02G530600	15.8	0.0	4.3 E‐18	8.0
CHI‐like	TraesCS5A02G146900	4.0	10.6	6.6 E‐08	3.1
CHI‐like	TraesCS5B02G145800	63.9	22.3	1.5 E‐07	2.6
CHI‐like	TraesCS5D02G145400	50.9	15.9	1.2 E‐08	2.8
F3H (TT6)	TraesCS2A02G493500	126.0	42.1	1.0 E‐13	2.8
F3H (TT6)	TraesCS2B02G521500	15.8	36.6	1.1 E‐16	3.6
F3H (TT6)	TraesCS2D02G493400	28.6	3.1	1.2 E‐15	5.4
F3'5'H	TraesCS6A02G369700	5.3	0.0	3.3 E‐12	5.8
F3'5'H	TraesCS6B02G405900	10.6	0.5	3.3 E‐15	6.3
F3'5'H	TraesCS6B02G406400	1.3	0.3	7.3 E‐18	7.5
DFR (TT3)	TraesCS3A02G226600	38.4	0.5	1.0 E‐25	10.3
DFR (TT3)	TraesCS3B02G257900	73.1	11.1	1.1 E‐24	5.1
DFR (TT3)	TraesCS3D02G224600	34.6	0.8	3.2 E‐28	11.1
LAR	TraesCS4A02G085900	6.5	0.0	6.8 E‐14	6.4
LAR	TraesCS4D02G218700	3.5	0.0	1.8 E‐03	2.7
GST (TT19/maize Bz2)	TraesCS7A02G374900	24.5	9.5	3.0 E‐03	2.2
R‐D1 (TT2/TaMyb10)	TraesCS3D02G468400	14.6	0.0	1.4 E‐19	8.6
MYBL2	TraesCS3A02G046800	4.9	0.0	4.7 E‐04	2.9

*Note*: Arabidopsis orthologues of wheat genes are in parentheses.

Abbreviations: TPM, transcripts per million; IP‐R and IP‐W, inner pericarp/seed coat of red and white NILs, respectively; *p*‐adj, adjusted *p*‐value; FC, fold change (R/W).

In contrast, no common theme could be discerned for any of the genes with higher expression in white grain, and the vast majority of such DEGs were not members of homoeologous series but singletons. Genes differentially expressed between red and white lines were also identified in the OP and E fractions: In OP, 35 genes were higher, and 77 were lower in expression (*p* < .05) in the red than the white NIL (Table S1), whereas in E, 66 genes were higher, and 86 were lower in expression in red (Table S3). Analysis of genes with higher expression in red OP tissue using KnetMiner showed that members of the *F3H* gene family were present, but transcript levels were much lower than in the IP fraction and are likely to be due to cross‐contamination of the tissue types during dissection. In contrast to the IP tissue, enrichment analysis of the genes differentially regulated in OP or E tissues of red versus white grain did not reveal any common themes or pathways.

It was noted that a disproportionate number of genes on chromosome 3B appeared to be more highly‐expressed in samples from red grain, irrespective of the tissue fraction (Tables [Link], [Link], [Link]): Out of a total of 147 genes higher in tissues from red grain, 37 (25.2%) are located on 3B, yet this chromosome contains only 5.7% of the genes identified in the Chinese Spring assembly (Appels et al., 2018). Furthermore, 25 of these genes are located in the distal region of the short arm of Chr 3B, within the first 10 Mbp of the pseudochromosome sequence (Figure S4). The most likely explanation is that the red and white NILs of cv. Holdfast differ in the complement of genes in this region of 3B. These genes in the distal region of Chr 3BS were therefore omitted from the functional analysis below.

### 3.3 Early biosynthetic genes (EBGs) in flavonoid biosynthesis

It has previously been shown that the early biosynthetic genes (EBGs) in proanthocyanidin biosynthesis, namely, *CHS*, *CHI*, and *F3H*, are more highly expressed in developing grain of red (R) versus white (r) isolines of wheat (Himi et al., 2005). *CHS* is represented by a small gene family in bread wheat, with at least 20 potential members including homoeologues, of which five members were found to be significantly (*p* < .05) more highly expressed in red grain of the Holdfast NILs (Tables 1 and S4), with increases ranging from 5‐ to 11‐fold. Phylogenetic analysis of the CHS‐related protein sequences suggested that the five differentially expressed genes fall into two paralogous families containing two and three homoeologues located on the group 2 chromosomes (Figure S5). In the case of *CHI*, bread wheat contains at least six genes in two paralogous families, both located on the group 5 chromosomes (Figure S6); all three homoeologues of one wheat CHI paralogue were more highly expressed in red IP tissues (Tables 1 and S4). This paralogue is more closely related to the Arabidopsis gene *At5G05270*, annotated as “chalcone isomerase‐like” (CHIL) than it is to *At3G55120* (*TT5*, encoding CHI). Wheat also contains a homoeologous set of three genes more closely related to Arabidopsis *TT5*/*CHI*, but although these are preferentially expressed in the IP tissue of developing grain, expression is not influenced by the presence of a functional *R* gene (Table S4 and Figure S6).

All three homoeologues of *F3H* were identified as more highly expressed in the red samples, confirming earlier observations in red‐ and white‐grained lines of wheat (Himi, Maekawa, & Noda, 2011). The *F3H* genes showed an average increase in red over white lines of 2.8‐fold and were predominantly expressed in the inner pericarp sample (Tables 1 and S4).

**FIGURE 5:**
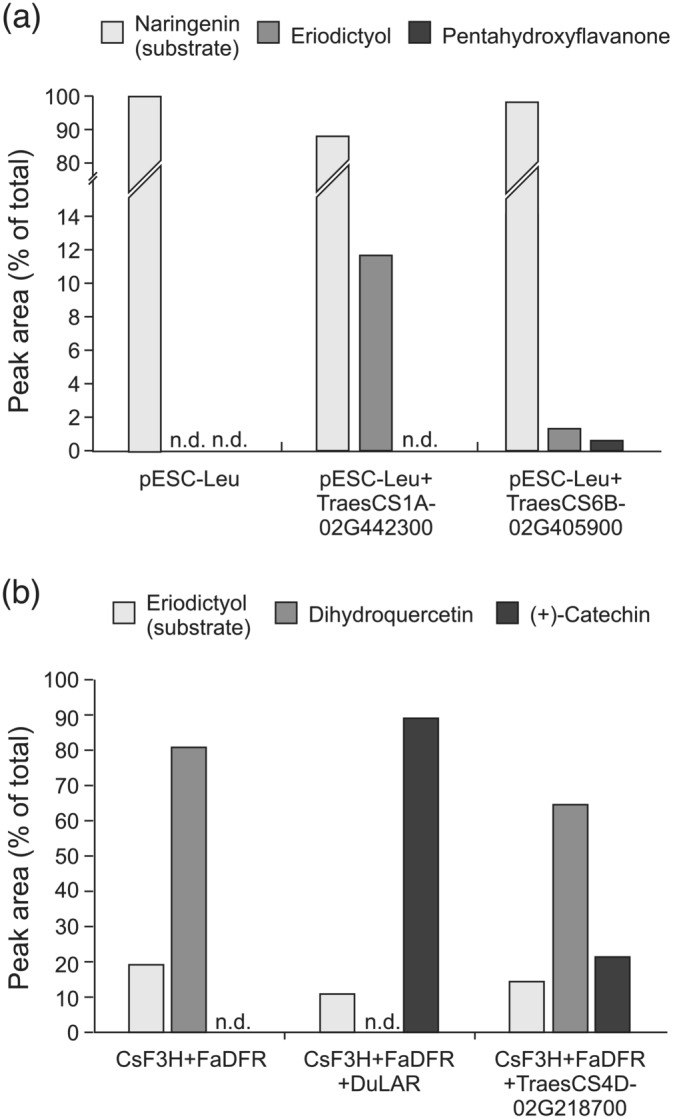
Heterologous expression of candidate PA genes. (a) Yeast cells expressing candidate wheat flavonoid 3′‐ and 3′,5′‐hydroxylases (TraesCS1A02G442300 and TraesCS6B02G405900) with naringenin as substrate. (b) *E. coli* cells co‐expressing a candidate LAR gene (TraesCS4D02G218700) together with F3H and DFR sequences, or LAR from *Desmodium uncinatum* as positive control, with eriodictyol as substrate. n.d., not detected

Genes encoding the cytochrome P450 monooxygenases flavonoid‐3′‐hydroxylase (*F3′H*) and the related flavonoid‐3′,5′‐hydroxylase (*F3′5′H*) have not previously been described in wheat. A BLAST search for sequences related to maize F3′H or F3′5′H identified a total of 15 sequences in the wheat TGACv1 proteome (Table S4). Of these, two groups, probably each forming a homoeologous set, are expressed in developing wheat grain: A triad of sequences on the long arms of the group 1 chromosomes are expressed in all three tissue fractions, albeit highest in IP, but not significantly differently between red and white grain (Table S4). Expression of a second group of four sequences on the long arms of the group 6 chromosomes is mainly confined to the IP, and three of the members were identified by DESeq2 as being significantly more highly expressed in red IP over white IP (Table 1 and highlighted in Table S4). It seems most likely that these two groups of related genes expressed in the developing grain encode F3′H and F3′5′H. Phylogenetic analysis of these together with other plant F3′H or F3′5′H sequences divided these related P450 sequences into two distinct clades (Figure S7), but as sequences annotated as F3′5′H appear in both clades, it was not possible to assign specific enzyme activities to the two wheat gene sets with any confidence. To resolve this, one candidate homoeologue from each set (TraesCS1A01G442300 and TraesCS6B01G405900) was expressed as a codon‐optimized synthetic cDNA in yeast. Induced yeast cultures were fed with substrate; cultures expressing TraesCS1A01G442300 converted naringenin to eriodictyol with an efficiency of 11.5%, demonstrating flavonoid 3′‐hydroxylase (F3′H) activity. Cells expressing TraesCS6B01G405900 converted naringenin to a mixture of eriodictyol and pentahydroxyflavanone (5′‐hydroxy‐eriodictyol) demonstrating sequential flavonoid 3′ and 5′ hydroxylase activities (Figure 5a). The low catalytic activity of heterologous expression products from TraesCS6B01G405900 may be indicative of poor expression, folding or membrane insertion in the yeast cells: for example, heterologous expression of F3′5′H from *Camellia sinensis* yielded minimal active enzyme until its N‐terminal signal peptide was replaced with that from *Vitis vinifera* F3′5′H (Wang et al., 2014). However, the evidence that expression products from TraesCS6B01G405900 can produce both eriodictyol and pentahydroxyflavanone from naringenin, albeit at low yield, together with phylogenetic analysis that clusters TraesCS6B01G405900 with the main clade of F3′5′H sequences (Figure S7) leads us to conclude that this gene encodes a flavonoid 3′,5′‐hydroxylase.

Although not previously classified as an EBG in the PA pathway of any species, the RNA‐Seq analysis also identified a group of three genes encoding phenyl ammonia lyase (PAL), located on the long arms of the group 2 chromosomes, as being 2.6‐ to 3.8‐fold more highly expressed in the IP of red grain, although transcripts were also present in the OP fraction of both the red and white NILs (Tables 1 and S4). PAL and the related PTAL (phenylalanine‐tyrosine ammonia lyase) are together encoded by a large multigene family in wheat, with more than 40 members; phylogenetic analysis of PAL‐PTAL homologues from wheat and rice indicated that the three differentially expressed genes are part of the PAL group and probably form a homoeologous triad on the long arms of the group 2 chromosomes (Figure S8). This raises the possibility that the supply of coumaroyl‐coA intermediate into the flavonoid pathway is promoted, directly or indirectly, by the R myb through increases in expression of a specific PAL paralogue. However, there was no evidence that any paralogues of later genes involved in the biosynthesis of coumaroyl‐coA, namely, cinnamate 4‐hydroxylase (*C4H*) and 4‐coumarate‐coA ligase (*4CL*), were more highly expressed in the IP of red lines (Table S4).

### 3.4 Late biosynthetic genes (LBGs) in flavonoid biosynthesis

Himi et al. (2005) reported that semi‐quantitative RT‐PCR of wheat *DFR* genes in developing grain of red and white near‐isogenic lines of wheat showed higher expression in the former. We similarly found that all three homoeologues of *DFR* were significantly more highly expressed in IP tissues of the red (*R‐D1b*) than the white (*R‐D1a*) NIL of cv. Holdfast, with DEseq2 reporting and average of 9‐fold higher transcript abundance in red grain (Tables 1 and S4). A lower level of expression was detected in red OP tissues, probably due to cross‐contamination with IP material during tissue isolation.

As shown in Figure 1, the proanthocyanidin biosynthetic pathway branches after leucocyanidin/leucodelphinidin to generate the alternative stereoisomers, (+)‐catechin and (−)‐epicatechin (gallocatechin and epigallocatechin if 5′‐hydroxylated), the terminal units of PA. We showed above (Figure 3) that wheat PA is composed exclusively of catechin with a small amount of gallocatechin; no epicatechin subunits were identified. In agreement with this, the RNA‐Seq analysis from developing wheat grain identified a homoeologous group of three genes with 53%–54% amino acid sequence identity with leucoanthocyanidin reductases from Spanish clover (*Desmodium uncinatum*) (*DuLAR*; Tanner et al., 2003, Figure S9) that were expressed in the IP tissue from developing red‐grained wheat; no expression was detected in white grain (Tables 1 and S4). To confirm that these candidates encoded functional LAR, one homoeologue (TraesCS4D01G218700) was co‐expressed in *E. coli* with cDNAs of flavanone 3‐hydroxylase (F3H) from *Camellia sinensis* and dihydroflavonol 4‐reductase (DFR) from *Fragaria ananassa* (Zhao et al., 2015). Induced cultures of bacteria harboring this plasmid converted eriodictyol via dihydroquercetin to (+)‐catechin (Figures 1 and 5b) with a product yield of 21.3%. This yield is lower than that produced by a similar plasmid expressing LAR from *Desmodium uncinatum* (Figure 5b) but within the range of yields achieved by heterologous expression using different combinations of F3H, DFR, and LAR genes described by (Zhao et al., 2015). This confirms that TraesCS4D01G218700 encodes a functional LAR from wheat.

In contrast, although five sequences related to anthocyanidin synthase (ANS or LDOX; Arabidopsis *TT18*) from the epicatechin branch of the pathway (Figure 1) were identified in the IWGSCv1.0 wheat assembly (Figure S10), only one of these was expressed at a significant level in developing grain, and predominantly in the OP tissue (Table S4). A large group of 30 genes on the group 2 chromosomes showed homology to anthocyanidin reductase (ANR) from Arabidopsis and other species (Figure S11). Several of these genes, including TraesCS4D01G218700 that shares 96% amino acid identity with a wheat sequence (SPT20169) shown to have ANR activity (Jun et al., 2021), were expressed in the tissues analyzed, but most highly in the OP sample and not differing in expression between the red and white isolines.

An additional component identified as a more highly expressed gene in the IP of red‐grained seeds, encoded by *TraesCS7A01G374900* (Tables 1 and S1), showed homology to maize *Bronze‐2* (*Bz2*) and Arabidopsis TRANSPARENT TESTA 19 (*TT19*), which encode glutathione *S*‐transferases potentially involved in flavonoid uptake into vacuoles. BLAST analysis identified two further wheat homoeologues of *TraesCS7A01G374900* that were also significantly more highly expressed in red IP than white IP, albeit at a level of significance below the false discovery threshold of *p* = .05 after correcting for multiple testing (Table S4). Phylogenetic analysis of the three wheat homoeologues together with ZmBz2 and AtTT19 in the context of related GST sequences from Arabidopsis and rice, which contains 56 members, showed that the wheat GSTs identified were more similar to maize *Bronze‐2*, from the Tau family of GSTs, than to Arabidopsis *TT19*, which lies within the Phi GST family (Figure S12).

In Arabidopsis, a number of additional genes involved in the later stages of PA biosynthesis have been identified (Table 1): These include *TT12* (*At3g59030*), encoding a MATE antiporter potentially involved in uptake of glycosylated PA monomers into the vacuole (Marinova et al., 2007). Wheat sequences homologous to TT12 were identified from the IWGSCv1.0 assembly and a phylogenetic tree constructed with 50 related sequences from Arabidopsis and 46 from rice; the relevant subclade is shown in Figure S13 and shows one clear orthologue of *TT12* in rice (*LOC_Os12g42130*) and two paralogues in wheat, each with three homoeologues, located on the short and long arms of the group 5 chromosomes. Although the developing grain RNA‐Seq data show that these six genes are most highly expressed in the inner pericarp fraction of developing grain, there was no significant difference in expression detected between red and white grains (Table S4). *TT13* (*At1g17260*) encodes a tonoplast ATPase (AHA10) that forms the vacuolar proton gradient required for flavonoid uptake (Appelhagen et al., 2015). BLAST searches and phylogenetic analysis identified single likely orthologues in rice (*LOC_Os03g08560*) and wheat, the latter with three homoeologues, located on wheat chromosome group 4 (Figure S14). As with *TT12*, the RNA‐Seq data show that the wheat *TT13* genes are most highly expressed in the Inner Pericarp fraction but not significantly influenced by the presence of a functional *R* gene (Table S4). Finally, a laccase, encoded by *TT10* (*At5g48100*), possibly involved in oxidative browning of PA has also been identified in Arabidopsis (Pourcel et al., 2005), but it proved impossible to identify unequivocally an orthologous group of sequences from wheat.

### 3.5 Transcription factors involved in flavonoid biosynthesis

A number of transcription factor genes are known to be involved in the control of the later PA‐biosynthetic genes in Arabidopsis. *TT2* (*At5g35550*) encodes a Myb and is the orthologue of the *R* Myb that determines grain color in wheat (Himi, Maekawa, Miura, & Noda, 2011). Of these, *TraesCS3D01G468400*, encoding R‐D1, also known as TaMyb10‐D is expressed in the IP tissues and is identified as a differentially expressed gene (Tables 1 and S4) due to deletion of the entire locus in the white NIL (Himi, Maekawa, Miura, & Noda, 2011). Its homoeologue *R‐B1* (*TraesCS3B01G515900*) is highly expressed in the IP samples of both red and white NILs: Both isolines contain a non‐functional allele, *R‐B1a*, that has a 19 bp deletion in exon 3. Both red and white lines also carry a loss‐of‐function allele of R‐A1 with a deletion of the promoter and first exon, but this is not annotated in the IWGSCv1.0 assembly and so not included in the transcriptome data. In addition to TT2, the heterotrimeric complex that regulates the late flavonoid genes of PA biosynthesis in Arabidopsis includes TT8 (At4g09820), a bHLH protein, and TTG1 (At5g24520), a WD40 (Baudry et al., 2004). A search for genes orthologous to *TT8* in wheat identified two likely homologues (TraesCS1A02G102400 and TraesCS1B02G112900) on the group 1 chromosomes (Figure S15), with highest expression in the IP tissue, and at similar levels in red and white NILs. We also identified two homoeologues (TraesCS6A02G259400 and TraesCS6B02G286700) of a candidate orthologue of TTG1, although sequence similarity between different WD40 proteins from Arabidopsis, rice and wheat proved too low to allow the generation of a meaningful phylogenetic tree. The candidate TTG1 homologues are expressed in all three developing grain tissues, but their expression is not affected by the presence of the *R* gene (Table S4).

In addition to the transcription factors above known to be involved in PA biosynthesis in Arabidopsis, two further wheat TFs were identified as significantly differentially expressed in the IP tissue of the red and white NILs. Expression of *TraesCS3A01G046800*, which phylogenetic analysis suggested is the orthologue of Arabidopsis MybL2 (*At1g71030*) (Figure S16), a transcription factor involved in control of flavonoid biosynthesis (Dubos et al., 2008), is confined to the IP and is approximately 3‐fold higher in the red NIL than the white (Table 1). The two likely homoeologues of *TraesCS3A01G046800* are also expressed only in the red IP tissues but with a non‐significant adjusted *p*‐value from DESeq2 (Table S4). The differential expression analysis also identified all three homoeologues of a WRKY‐class transcription factor (Tables S1 and S4), most closely related to the rice protein OsWRKY23 (Figure S17), that were 3‐ to 6‐fold over‐expressed in IP tissue of the red NIL and therefore may also play a role in control of the pathway.

## 4 DISCUSSION

The red pigment in the true seed coat (testa) of wheat grains influences the agronomic performance of the crop, through its role in providing resistance to pre‐harvest sprouting, and is also a determinant of grain quality: White lines that lack the pigment have a higher extraction rate of white flour as contamination with bran particles has less influence on flour color, and whole grain flour from white wheat has a less astringent taste than flour from red lines. By analogy with barley, it was predicted that the seed coat pigment in wheat was proanthocyanin, and McCallum and Walker (1990) detected small amounts of catechin and PA oligomers in the bran of mature wheat grain. More recently, Kohyama et al. (2017) identified catechin and PA oligomers in developing wheat grain at 10 days post anthesis. In this work, we show that PAs from wheat grain are composed exclusively of polymers of (+)‐catechin, with a small proportion of gallocatechin (5′‐hydroxycatechin) subunits (Figures 1 and 3); no (−)‐epicatechin was identified as monomers or in polymers, in contrast to Arabidopsis, whose seed coat PA is composed entirely of epicatechin submits (Abrahams et al., 2003). We also show that the mean degree of polymerization of wheat grain PA increases between 10 and 20 days post anthesis (Figure 4) and that insoluble PAs—those not extractable with 70% acetone—have a higher degree of polymerization. This may explain our observation that bran from mature wheat grain, in contrast to barley, contains no extractable PA monomers or oligomers, although further modification and/or cross‐linking to other cell components that prevent solubilization cannot be excluded.

Himi and colleagues previously characterized the early genes of PA biosynthesis in wheat (*CHS*, *CHI*, and *F3H*), together with *DFR* and showed that these were all strongly upregulated in red grain, that is, grain with a functional R Myb in maternal tissues, compared with white grain (Himi et al., 2005). Here, we show that a comparison of the transcriptome of the “inner pericarp” tissue of developing grains from red (*R‐A1a*/*R‐B1a*/*R‐D1b*) and white (*R‐A1a*/*R‐B1a*/*R‐D1a*) NILs of bread wheat cv. Holdfast exhibited differential expression of the four genes above and also identified further candidates encoding components of the PA biosynthetic pathway that are controlled, directly or indirectly, by the R Myb. These include genes encoding a paralogue of phenyl ammonia lyase (PAL); a gene shown to encode flavonoid 3′,5′‐hydroxylase (F3′5′H); leucoanthocyanidin reductase (LAR) and a glutathione *S*‐transferase related to maize *Bronze‐2* and Arabidopsis *TT19* (Table 1). Analysis of genes up‐ or down‐regulated in other tissues of the red NIL using KnetMiner (Hassani‐Pak et al., 2016) did not reveal any common themes, with the exception of a group of genes in the telomeric region of chromosome 3B that were all more highly expressed in all Red tissues (Figure S4), most easily explained as a large deletion in this region of 3BS in the white parental line compared to Chinese Spring, the donor of the wild‐type *R‐D1b* allele, that has not been resolved by the backcrossing to BC_6_ that created the red NIL in the white Holdfast background (Bassoi & Flintham, 2005).

In addition to the *R* gene previously shown to encode a MYB orthologous to Arabidopsis *TT2* (Himi & Noda, 2005), we identified likely orthologues of the bHLH and WD40 proteins that in Arabidopsis form a heterotrimeric complex that controls the LBGs. Expression of the homoeologues of *R* and of the bHLH, orthologous to *TT8*, was mainly confined to the IP tissue samples containing the inner integument cell in which PAs accumulate (Table S4 and Figure 2), whereas the candidate orthologue of *TTG1* was expressed in all tissues. Further work will be required to demonstrate that these transcription factors play a role in controlling the expression of flavonoid genes in the PA pathway.

Although the data above do not provide evidence for direct control of flavonoid genes by the R Myb, it is interesting that the presence of a functional *R* gene increases expression of both early (*CHS*, *CHI*, and *F3H*) and late (*DFR*, *F3′5′H*, *LAR*, and *Bz2/TT19*) biosynthetic genes in the wheat pathway. This is in contrast to Arabidopsis seed tissues, where a ternary complex of a MYB (TT2 or MYB5), a bHLH (TT8 or EGL3), and a WDR protein (TTG1) directly controls only the later genes, in this case including TT12 (a MATE transporter) and TT13 (a proton‐ATPase) (Xu et al., 2014). Thus, loss of function of Arabidopsis *TT2*, the orthologue of wheat *R*, does not affect expression of *CHS*, *CHI*, or *F3H*. Strikingly, we found that all three homoeologues of a specific paralogue of *PAL*, from the start of the phenylpropanoid pathway, were also up‐regulated in IP tissues of the red NIL. This is reminiscent of observations in poplar, where overexpression of the *TT2* orthologue, PtMYB134, promoted expression of multiple genes in the phenylpropanoid pathway including PAL, 4CL, CHS, CHI, DFR, ANS, ANR, and LAR (Mellway et al., 2009).

Above, we showed that wheat grain PA is composed exclusively of (+)‐catechin or (+)‐gallocatechin as both the terminal and extension unit. This is reflected in the expression of the late PA pathway genes, where there is induction of the A and B homoloeogues of LAR (the D homoeologue is not annotated in the IWGSCv1.0 assembly) in the IP tissue of the red‐grained NIL, indicating direct or indirect control by the R MYB. LAR catalyzes production of the PA terminal (+)‐catechin unit from leucocyanidin (Figure 1); paralogues of ANR, which in other species is capable of producing both (+)‐catechin and (−)‐epicatechin, are also expressed in the grain tissues, but most highly in the OP tissue and not affected by the presence of the R Myb that controls PA biosynthesis in the IP; in addition, we did not detect (−)‐epicatechin, a major product of ANR activity, in the wheat grain tissues (Figure 3). It seems most likely, therefore, that the (+)‐catechin required for wheat grain PA biosynthesis is produced by LAR. However, the details of PA assembly from the catechin terminal are still somewhat unclear. Recent work in *Medicago trunculata*, which accumulates PAs containing (−)‐epicatechin, indicates that the starter and extension units of *Medicago* PA are derived from different branches of the pathway from leucocyanidin: the terminal epicatechin unit is produced from (+)‐catechin via flav‐2‐en‐3‐ol by the enzymes LDOX and ANR while the addition unit is probably derived from 2,3‐cis‐leucocyanidin by epimerization of 2,3‐cis‐leucocyanidin by ANS and a second function of ANR (Jun et al., 2021). Interestingly, a *Medicago* mutant lacking both ANS and LDOX makes PAs consisting of (+)‐catechin terminal and extension units; it is assumed that the terminal catechin is derived from leucocyanidin through the action of LAR, whereas the best candidate for the extension unit is derived from 2,3‐trans‐leucocyanidin (see Figure 5 in Jun et al., 2021). This scheme may be a good model for understanding the assembly of wheat and barley PAs and could be tested by manipulating levels of LAR, which would vary the availability of the terminal units and thereby should modify the chain length of the PA polymer and, potentially, its solubility.

A long‐term aim of this project is to investigate the link between grain color and pre‐harvest sprouting in wheat in order to increase the range of environments suitable for white wheat production. The observation that in many species, including wheat, loss of seed coat PA due to mutations in structural or regulatory genes from the biosynthetic pathway results in premature germination implies that PA contributes to dormancy. However, the Ant17 mutant of barley, which contains a premature stop codon in the F3H gene, lacks both anthocyanin and proanthocyanidin but is reported to retain the same level of seed dormancy as wild‐type seeds (Himi & Taketa, 2015). This provides some encouragement that manipulation of the PA pathway in wheat by TILLING or CRISPR/Cas9, enabled by the identification of the genes from the pathway in this work, could generate wheat lines with reduced PA content without compromising resistance to pre‐harvest sprouting.

## CONFLICTS OF INTEREST

The authors did not report any conflicts of interest.

## AUTHOR CONTRIBUTIONS

A.L.P., A.K.H., and S.P.V. conceived and designed the work; J.M.B. and J.L.W. developed and provided analytical chemistry methods; L.P. and A.P. contributed the bioimaging and S.P.V. and A.K.H produced the dissected grain tissues and RNA samples which were sequenced by J.C. Bioinformatics analysis was provided by A.L.P., R.K., and K.H.‐P. A.L.P. and S.K. carried out the heterologous expression. A.L.P. wrote the manuscript.

## Supporting information


**Table S1:** Differentially expressed genes in the “outer pericarp” (OP) tissues of developing grains of red vs white NILs.

**Table S2:** Differentially expressed genes in the “inner pericarp” (IP) tissues.

**Table S3:** Differentially expressed genes in the endosperm (E) tissue.

**Table S4:** Expression data for PA biosynthetic genes in OP, I and E tissues.

**Figure S1**: ESI‐MS analysis of wheat and barley PAs.
**Figure S2**: Chemistry of thiolysis of PAs
**Figure S3**: Smear plots from the analysis of differential expression
**Figure S4**: Differentially expressed genes on the Group 3 chromosomes.
**Figures S5–S17**: Phylogenetic trees of protein sequences of PA candidate genes. Figure S5: CHS; Figure S6: CHI; Figure S7: F3′H/F3′5′H; Figure S8: PAL/PTAL; Figure S9: LAR; Figure S10: ANS/FLS/LDOX; Figure S11: ANR; Figure S12: TT19/Bronze‐2; Figure S13: TT12/MATE; Figure S14: TT8/bHLH; Figure S15: MybL2 Figure S16: TT13/AHA10; Figure S17: WRKY23 homologues

## Data Availability

The RNA‐seq data that support the findings of this study are openly available in the European Nucleotide Archive at http://ebi.ac.uk/ena, accession number E‐MTAB‐9006.
