# Supplementary material for: Proanthocyanidin biosynthesis in the developing wheat seed coat investigated by chemical and RNA‐Seq analysis
Source: Plant Direct. 2022 Oct 12;6(10):e453. doi: 10.1002/pld3.453 (PMC9554643; doi:10.1002/pld3.453)
Supplement: Supplementary file 5 — Figure S1: ESI‐MS analysis of wheat and barley PAs. Figure S2: Chemistry of thiolysis of PAs Figure S3: Smear plots from the analysis of differential expression Figure S4: Differentially expressed genes on the Group 3 chromosomes. Figures S5–S17: Phylogenetic trees of protein sequences of PA candidate genes. Figure S5: CHS; Figure S6: CHI; Figure S7: F3′H/F3′5′H; Figure S8: PAL/PTAL; Figure S9: LAR; Figure S10: ANS/FLS/LDOX; Figure S11: ANR; Figure S12: TT19/Bronze‐2; Figure S13: TT12/MATE; Figure S14: TT8/bHLH; Figure S15: MybL2 Figure S16: TT13/AHA10; Figure S17: WRKY23 homologues [file PLD3-6-e453-s003.pdf]

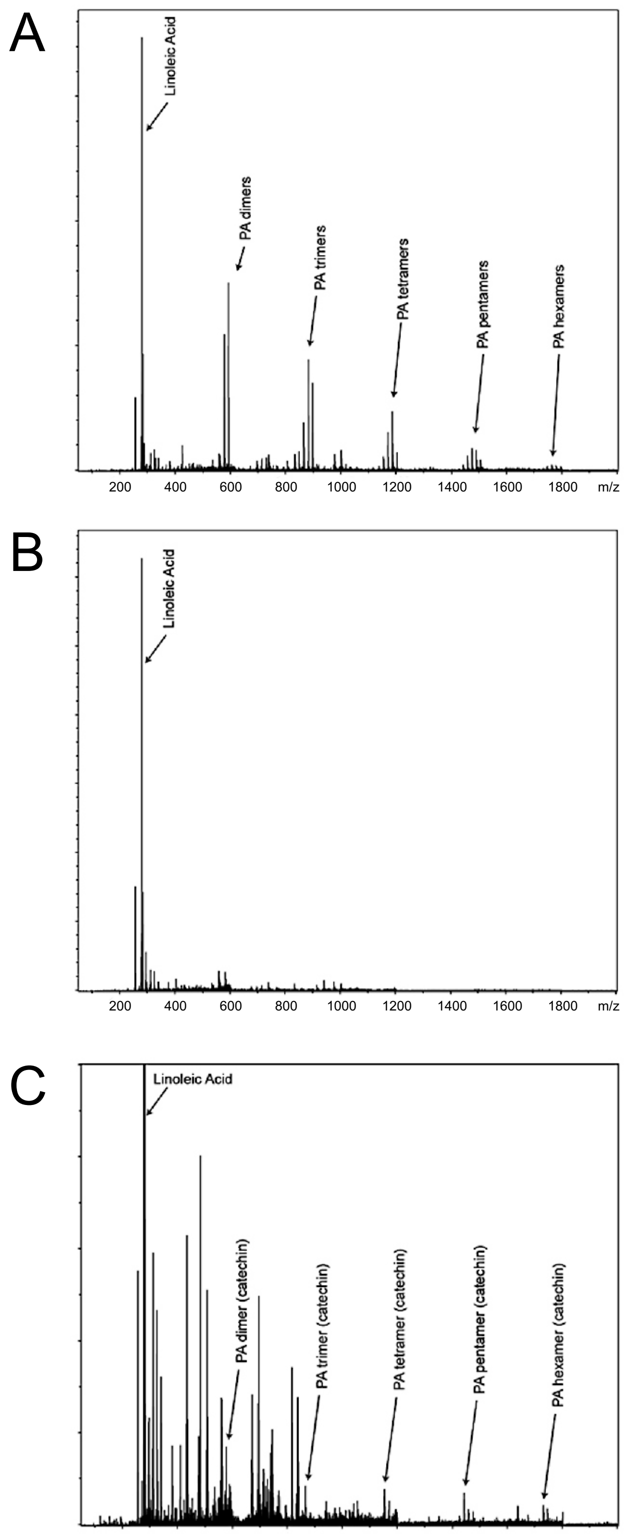

Supplementary Figure S1: Negative mode ESI-MS spectra of 70% acetone extracts of barley and wheat grain identifying oligomers of proanthocyanidin (PA). A: Mature barley caryopses. B: Mature wheat caryopses. C: Developing grain from wheat at 15 days post-anthesis.

A

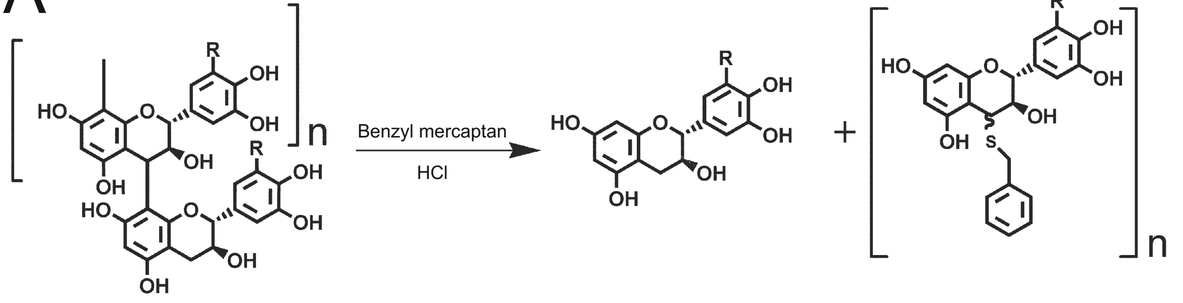

Proanthocyanidin
R=H (4->8)-linked catechin
R=OH 4->8-linked gallocatechin

Terminal unit
R=H catechin
R=OH gallocatechin

Extension unit
R=H catechin benzylthioether
R=OH gallocatechin benzylthioether
(Mixture of 3,4-*trans* and 3,4-*cis*)

B

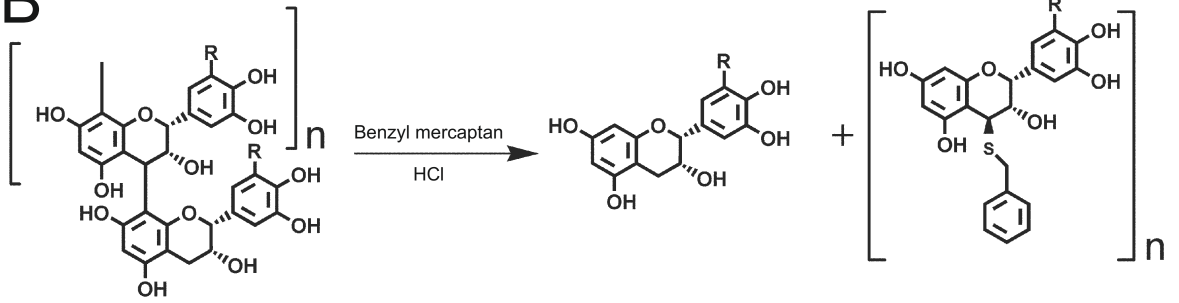

Proanthocyanidin
R=H (4->8)-linked epicatechin
R=OH 4->8-linked epigallocatechin

Terminal unit
R=H epicatechin
R=OH epigallocatechin

Extension unit
R=H epicatechin benzylthioether
R=OH epigallocatechin benzylthioether
(exclusively 3,4-*trans*)

Supplementary Figure S2: Thiolysis of polymeric PAs with acidic benzyl mercaptan results in the production of the terminal unit (catechin or epicatechin), and *n* copies of the addition unit as benzylthioether conjugates, that retain the stereochemistry at C2-C3. (A) Catechin addition units are released as a mixture of 3,4-cis and 3,4-trans benzylthioethers, whereas (B) epicatechin addition units form only 3,4-trans conjugates.

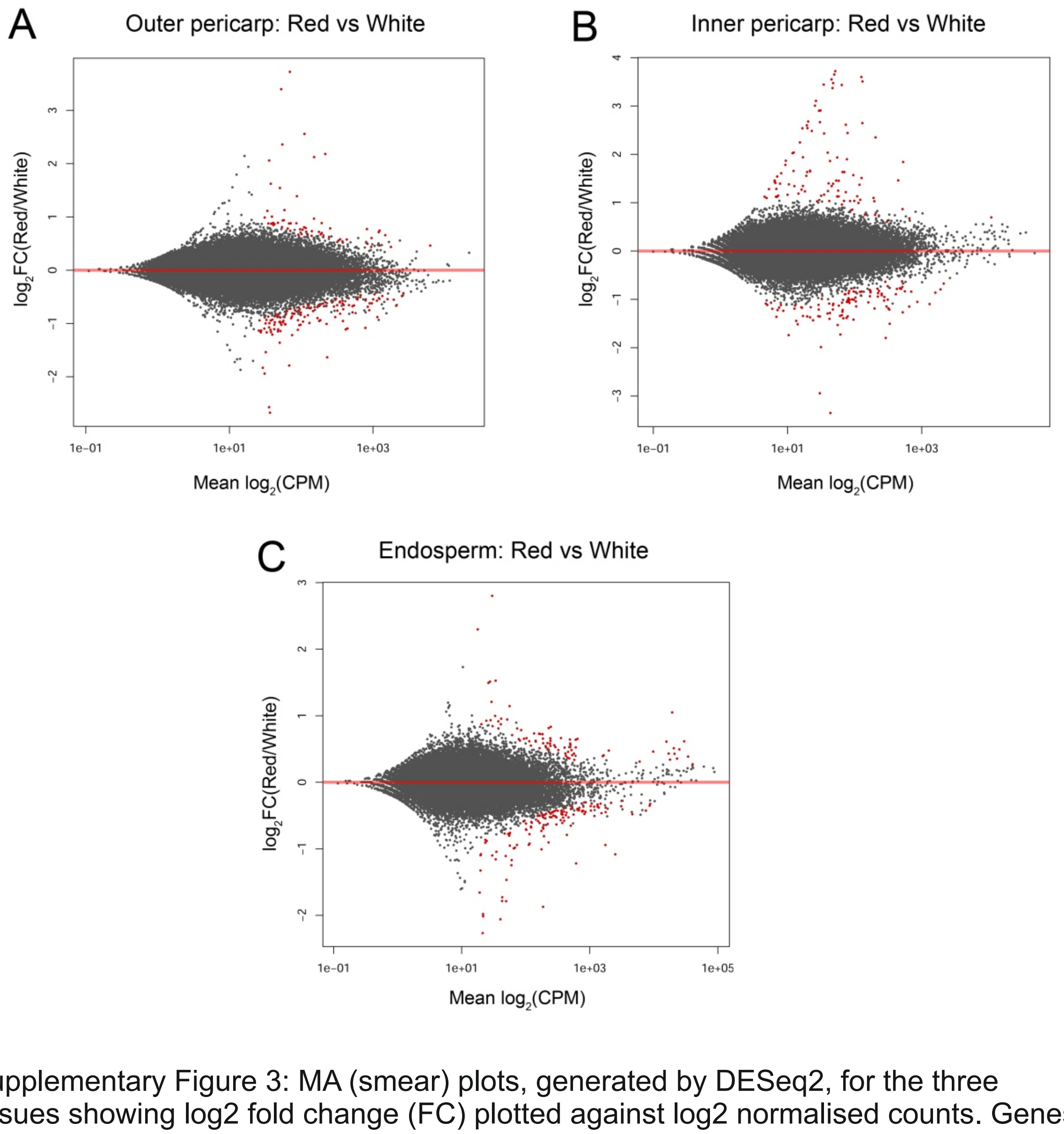

Supplementary Figure 3: MA (smear) plots, generated by DESeq2, for the three tissues showing log2 fold change (FC) plotted against log2 normalised counts. Genes in red have an adjusted p-value <0.1.

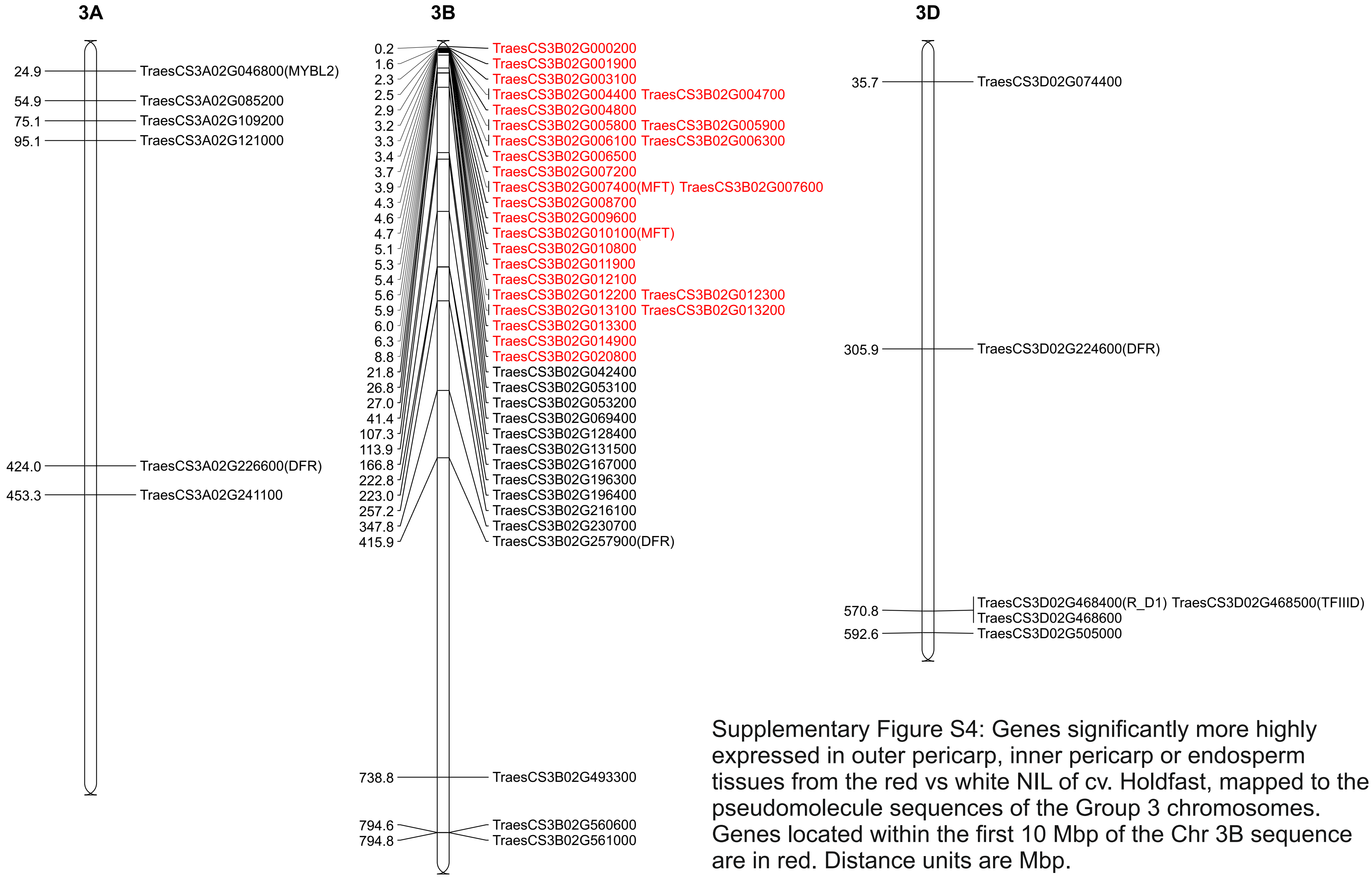

Supplementary Figure S4: Genes significantly more highly expressed in outer pericarp, inner pericarp or endosperm tissues from the red vs white NIL of cv. Holdfast, mapped to the pseudomolecule sequences of the Group 3 chromosomes. Genes located within the first 10 Mbp of the Chr 3B sequence are in red. Distance units are Mbp.

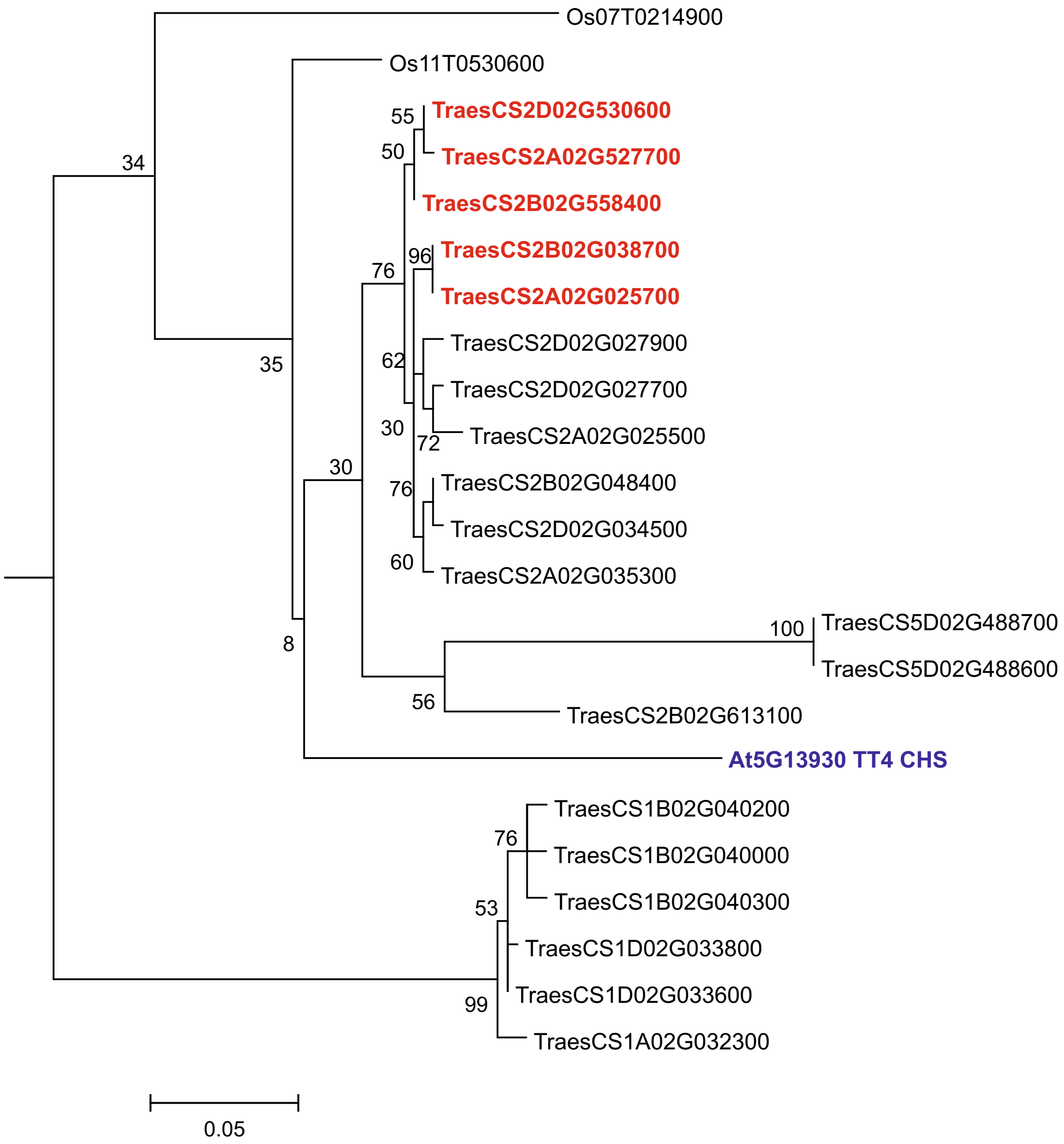

Supplementary Figure S5: Maximum likelihood (ML) tree of wheat (Traes), rice (Os) and Arabidopsis (At) sequences related to chalcone synthase (CHS). Genes significantly more highly expressed in inner pericarp tissue of the red vs white NIL are in red. Branch lengths denote amino acids substitutions per position, shown in the scale bar; branch support values are from 100 bootstrap runs.

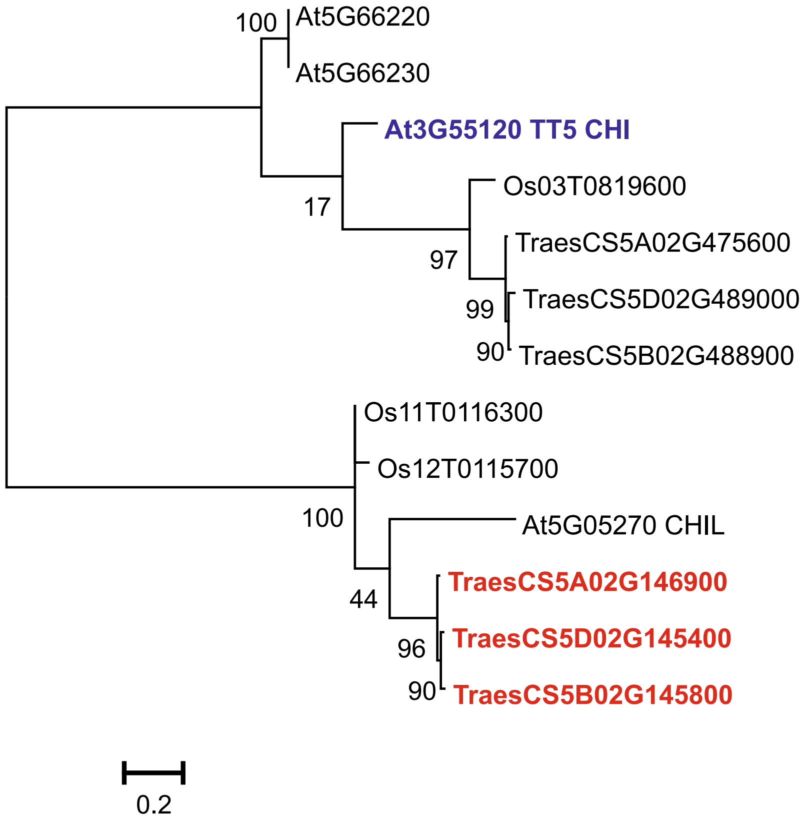

Supplementary Figure S6: Maximum likelihood (ML) tree of wheat (Traes), rice (Os) and Arabidopsis (At) sequences related to chalcone isomerase (CHI). Genes significantly more highly expressed in inner pericarp tissue of the red vs white NIL are in red. Branch lengths denote amino acids substitutions per position, shown in the scale bar; branch support values are from 100 bootstrap runs.

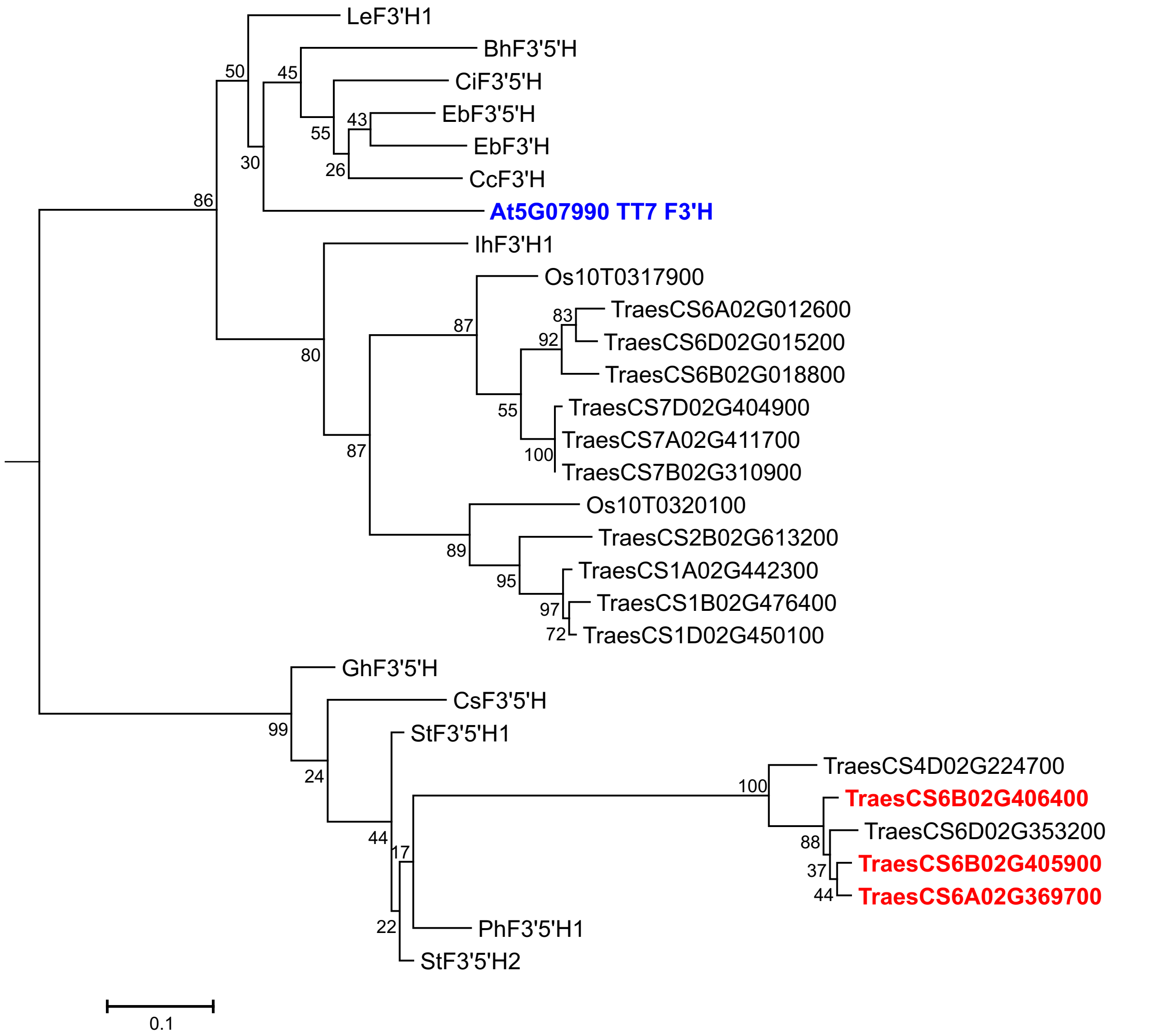

Supplementary Figure S7: Maximum likelihood (ML) tree of sequences related to flavonoid 3'-hydroxylase (F3'H) and flavonoid 3',5'-hydroxylase (F3'5'H) from wheat (Traes), Arabidopsis (At), rice (Os) and other species: *Centaurea cyanus* (Cc), *Cichorium intybus* (Ci), *Camellia sinensis* (Cs), *Echinops bannaticus* (Eb), *Gossypium hirsutum* (Gh), *Iris x hollandica* (Ih), *Lobelia erinus* (Le), *Petunia x hybrida* (Ph), *Zea mays* (Zm). Wheat genes significantly more highly expressed in inner pericarp tissue of the red vs white NIL are in red. Branch lengths denote amino acids substitutions per position, shown in the scale bar; branch support values are from 100 bootstrap runs.

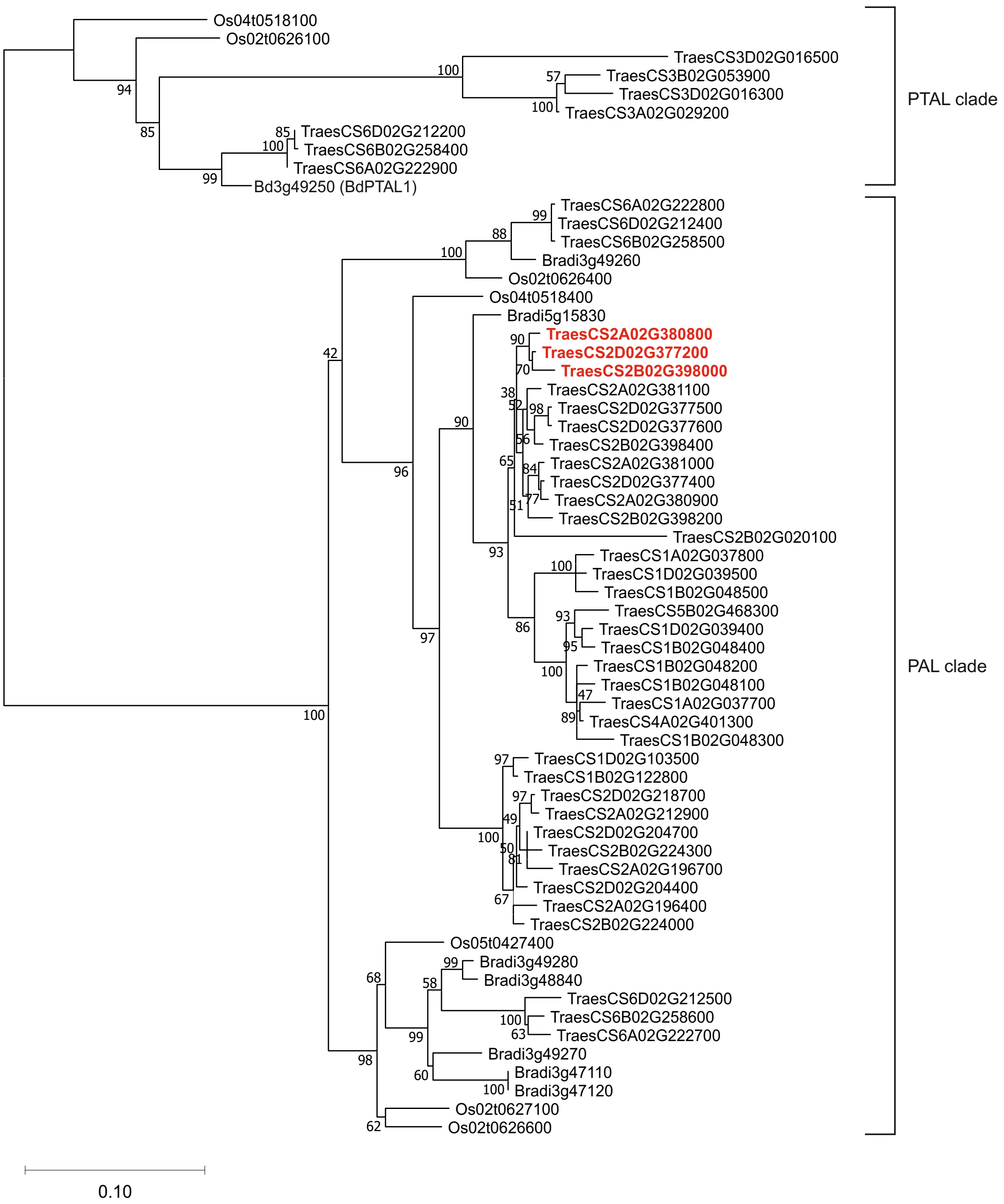

Supplementary Figure S8: Maximum likelihood (ML) unrooted tree of bread wheat (Traes), *Brachypodium distachyon* (Bd) and rice (Os) sequences related to phenylalanine ammonia lyase (PAL) and bi-functional phenylalanine/tyrosine ammonia lyase (PTAL). Wheat genes significantly more highly expressed in inner pericarp tissue of the red vs white NIL are in red. Branch lengths denote amino acids substitutions per position, shown in the scale bar; branch support values are from 100 bootstrap runs.

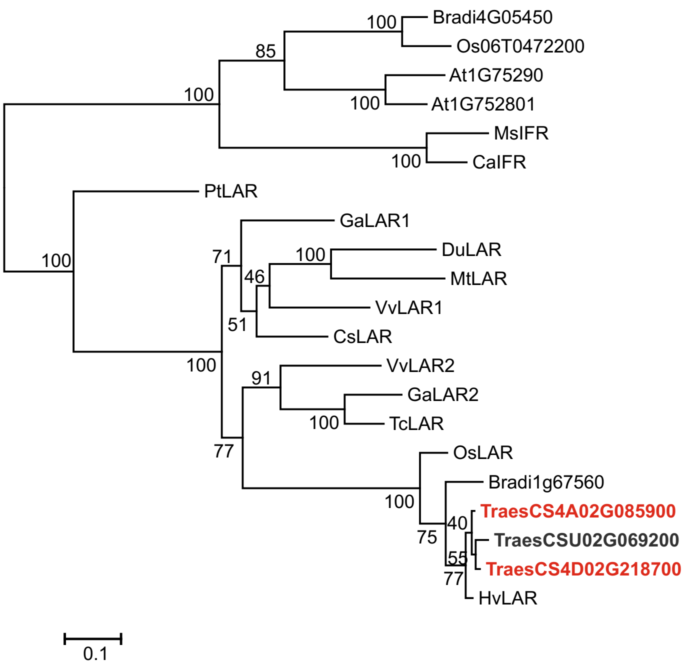

Supplementary Figure S9: Maximum likelihood (ML) tree of protein sequences related to leucoanthocyanidin reductase (LAR) and isoflavone reductase (IFR) from wheat (Traes), *Brachypodium distachyon* (Bradi), rice (Os), cocoa (Tc), cotton (Ga), grapevine (Vv), tea (Cs), *Medicago trunculata* (Mt), *Desmodium uncinatum* (Du), Loblolly pine (Td), *Medicago sativa* (Ms) and Arabidopsis (At). Wheat genes significantly more highly expressed in inner pericarp tissue of the red vs white NIL are in red. Branch lengths denote amino acids substitutions per position, shown in the scale bar; branch support values are from 100 bootstrap runs.

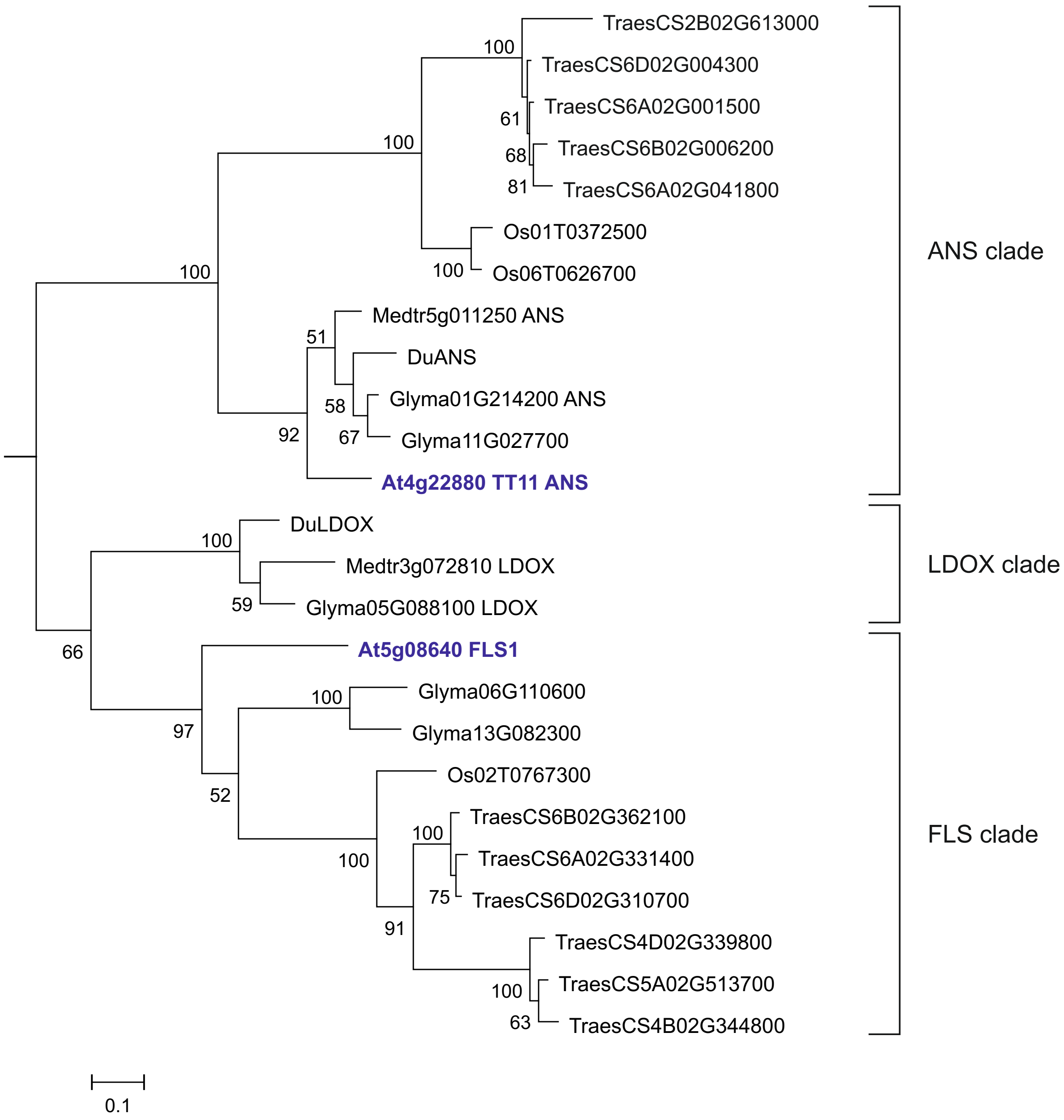

Supplementary Figure S10: Maximum likelihood (ML) tree of wheat (Traes) sequences related to anthocyanidin synthase (ANS), leucoanthocyanidin dioxygenase (LDOX) or flavonol synthase (FLS). Sequences from Arabidopsis (At), rice (Os), *Medicago truncatula* (Medtr), and *Glycine max* (Glyma) were identified in Phytozome. Sequences from *Desmodium uncinatum* were from Jun et al. 2018. The tree was rooted with At4g10490 (flavone synthase) Branch lengths denote amino acids substitutions per position, shown in the scale bar; branch support values are from 100 bootstrap runs.

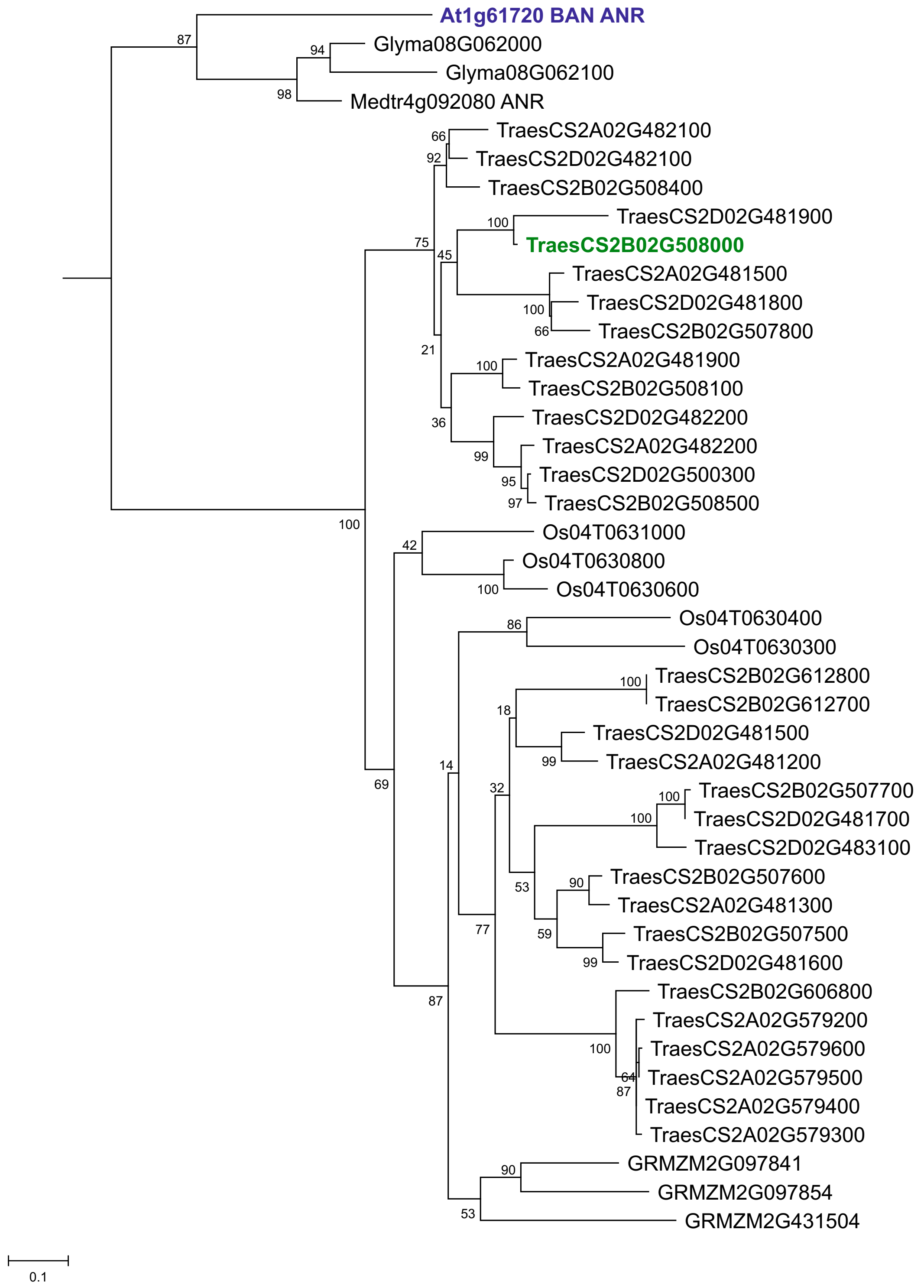

Supplementary Figure S11: Maximum likelihood (ML) tree of wheat (Traes) sequences related to anthocyanidin reductase/Banyuls (ANR/BAN). Sequences from Arabidopsis (At), rice (Os), *Medicago trunculata* (Medtr), and *Glycine max* (Glyma) were identified in Phytozome. The tree was rooted with At5g42800 (dihydroflavonol reductase). The wheat and barley ANR sequences studied by Jun et al. (2021) are most closely related (96% and 93% identity, respectively) to the sequence TraesCS2B02G508000 highlighted in green. Branch lengths denote amino acids substitutions per position, shown in the scale bar; branch support values are from 100 bootstrap runs.

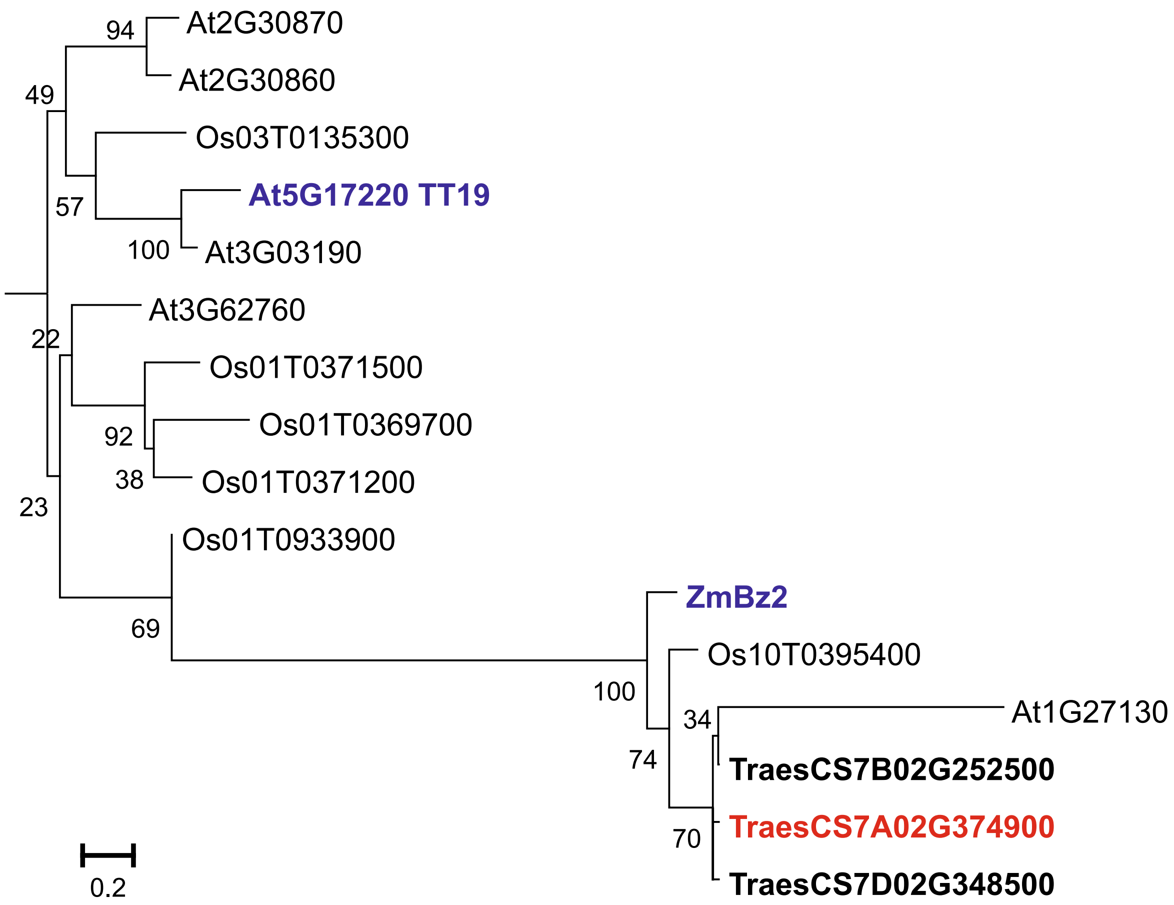

Supplementary Figure S12: Maximum likelihood (ML) tree of wheat (Traes), rice (Os) and Arabidopsis (At) sequences related to maize Bronze-2 (ZmBz2) and Arabidopsis TRANSPARENT TESTA 19 (TT19; At5g17220). Wheat genes significantly more highly expressed in inner pericarp tissue of the red vs white NIL are in red. Branch lengths denote amino acids substitutions per position, shown in the scale bar; branch support values are from 100 bootstrap runs.

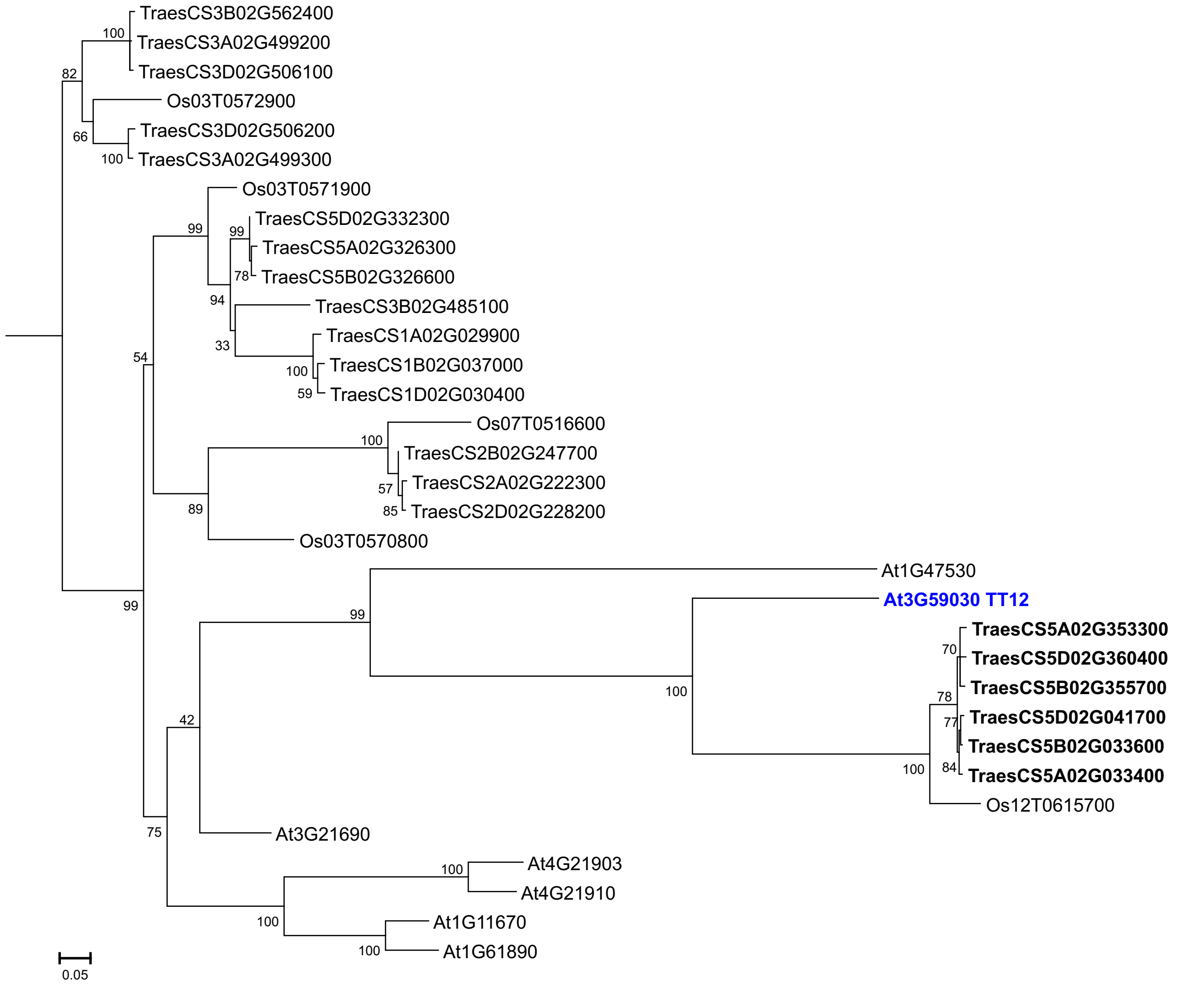

Supplementary Figure S13: Maximum likelihood (ML) tree of wheat (Traes), rice (Os) and Arabidopsis (At) sequences related to the Arabidopsis MATE transporter, TT12. Branch lengths denote amino acids substitutions per position, shown in the scale bar; branch support values are from 100 bootstrap runs.

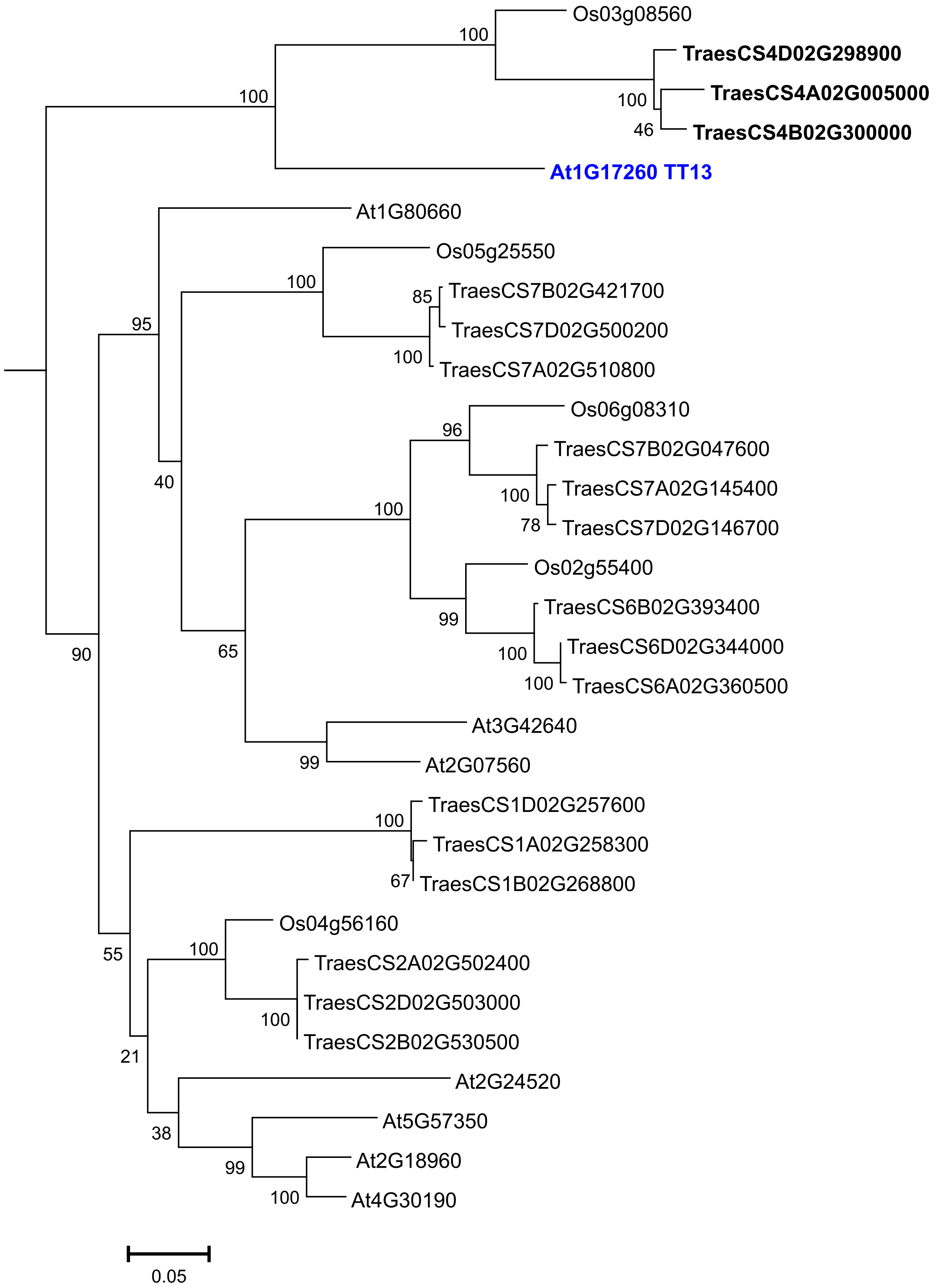

Supplementary Figure S14: Maximum likelihood (ML) tree of wheat (Traes), rice (Os) and Arabidopsis (At) sequences related to Arabidopsis TT13/AHA10, encoding a tonoplast proton-ATPase. Branch lengths denote amino acids substitutions per position, shown in the scale bar; branch support values are from 100 bootstrap runs.

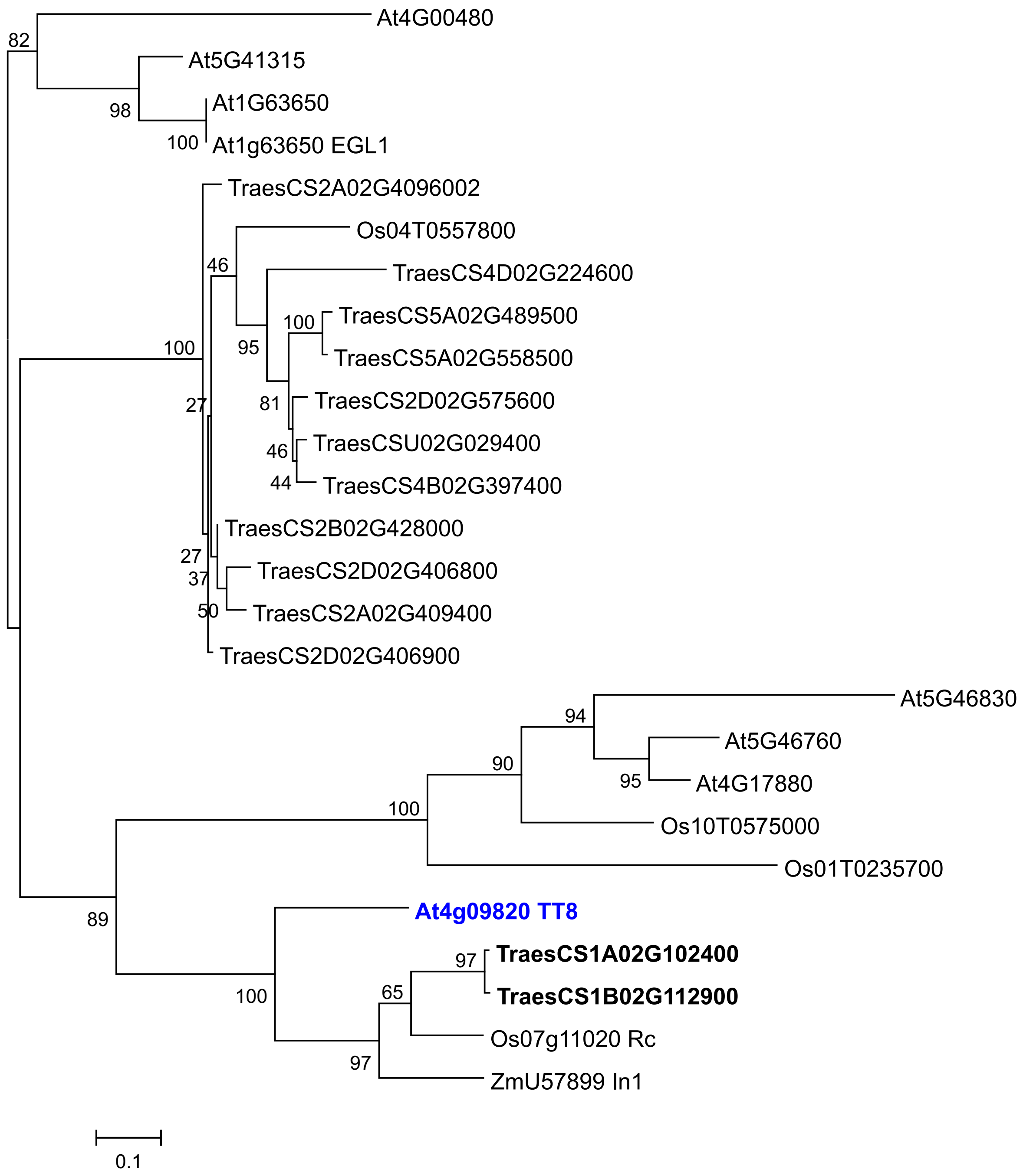

Supplementary Figure S15: Maximum likelihood (ML) tree of wheat (Traes), rice (Os), maize (Zm) and Arabidopsis (At) sequences related to Arabidopsis TT8, encoding a bHLH transcription factor. Branch lengths denote amino acids substitutions per position, shown in the scale bar; branch support values are from

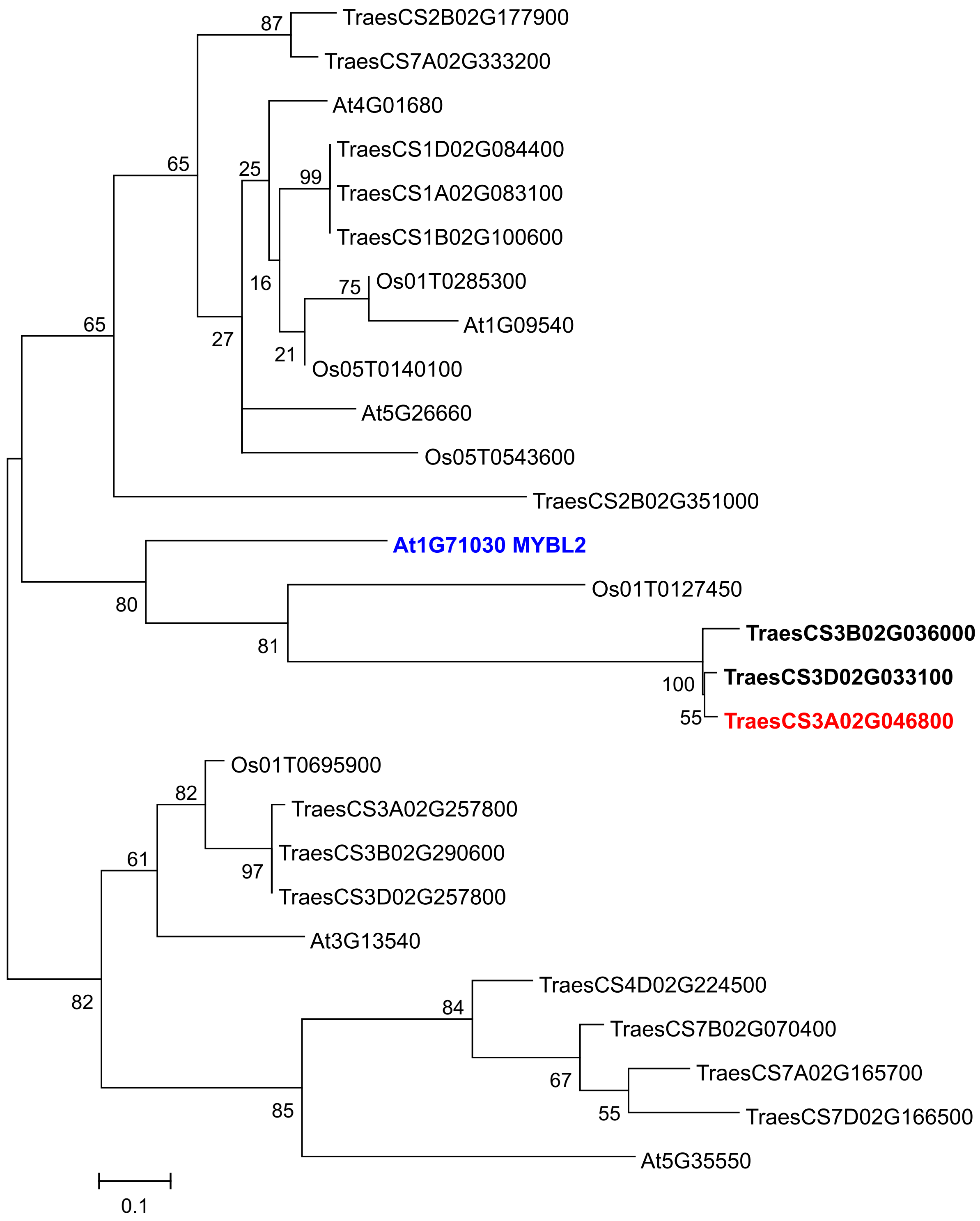

Supplementary Figure S16: Maximum likelihood (ML) tree of wheat (TraesCS), rice (Os) and Arabidopsis (At) sequences related to AtMybL2. Branch lengths denote amino acids substitutions per position, shown in the scale bar; branch support values are from 100 bootstrap runs. Genes differentially expressed in grain IP are in red.

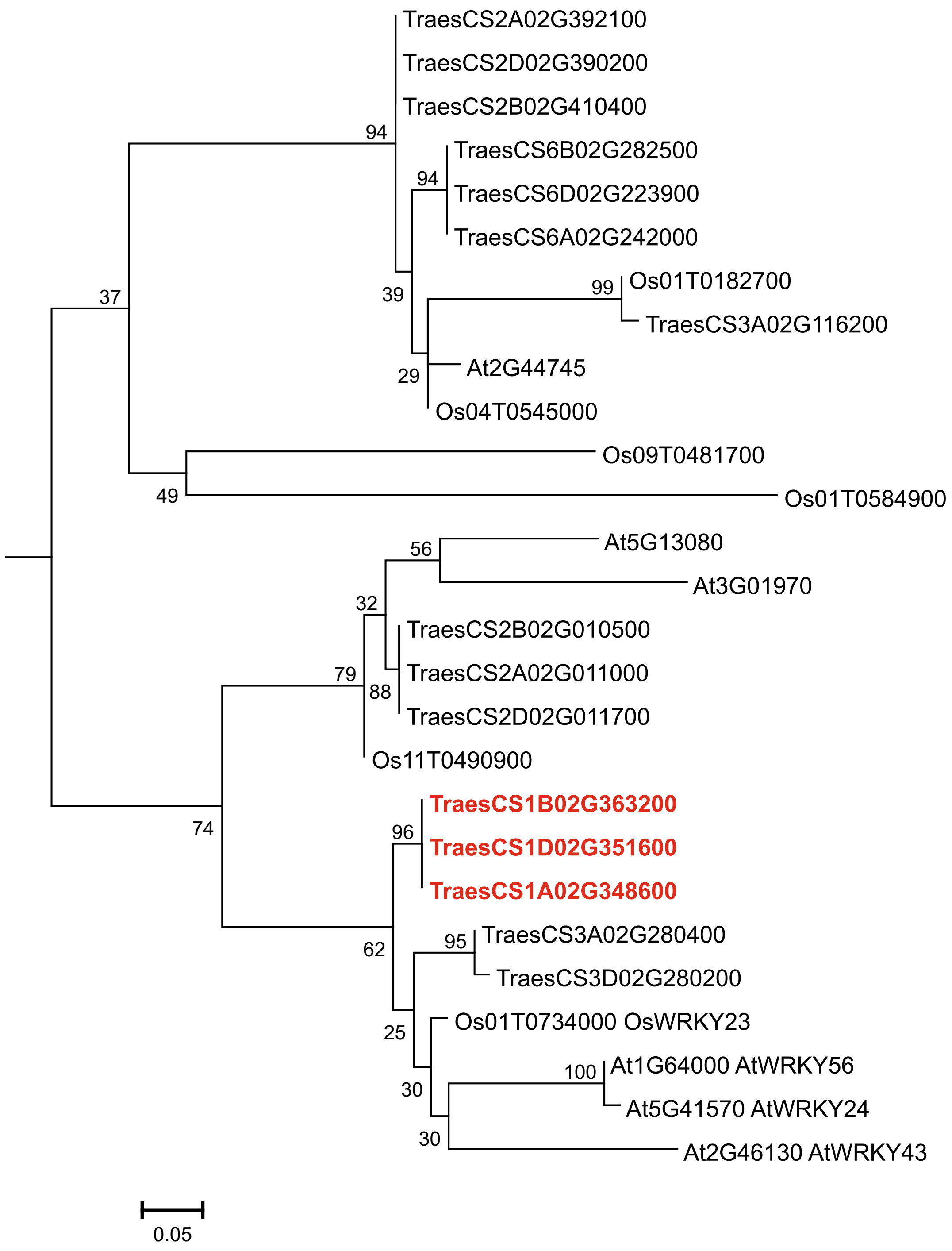

Supplementary Figure S17: Maximum likelihood (ML) tree of wheat (Traes), rice (Os) and Arabidopsis (At) sequences related to rice OsWRKY23. Branch lengths denote amino acids substitutions per position, shown in the scale bar; branch support values are from 100 bootstrap runs. Genes differentially expressed in the wheat grain IP tissue are in red.
